# Identification of a neural crest stem cell niche by Spatial Genomic Analysis

**DOI:** 10.1038/s41467-017-01561-w

**Published:** 2017-11-28

**Authors:** Antti Lignell, Laura Kerosuo, Sebastian J. Streichan, Long Cai, Marianne E. Bronner

**Affiliations:** 1 Division of Chemistry and Chemical Engineering, California Institute of Technology, Pasadena, CA 91125 USA; 2 Division of Biology and Biological Engineering, California Institute of Technology, Pasadena, CA 91125 USA; 3 Biomolecular Science and Engineering, University of California, Santa Barbara, Santa Barbara, CA 93106 USA

## Abstract

The neural crest is an embryonic population of multipotent stem cells that form numerous defining features of vertebrates. Due to lack of reliable techniques to perform transcriptional profiling in intact tissues, it remains controversial whether the neural crest is a heterogeneous or homogeneous population. By coupling multiplex single molecule fluorescence in situ hybridization with machine learning algorithm based cell segmentation, we examine expression of 35 genes at single cell  resolution in vivo. Unbiased hierarchical clustering reveals five spatially distinct subpopulations within the chick dorsal neural tube. Here we identify a neural crest stem cell niche that centers around the dorsal midline with high expression of neural crest genes, pluripotency factors, and lineage markers. Interestingly, neural and neural crest stem cells express distinct pluripotency signatures. This Spatial Genomic Analysis toolkit provides a straightforward approach to study quantitative multiplex gene expression in numerous biological systems, while offering insights into gene regulatory networks via synexpression analysis.

## Abstract (web summary)

Neural crest cells arise within the central nervous system, then migrate and contribute to a variety of cell types. Here, the authors use multiplex transcript analysis at single cell  resolution to define neural crest and neural subpopulations within the avian neural tube, including a neural crest stem cell niche.

## Introduction

A central question in developmental biology is how individual stem cells acquire the ability to differentiate into multiple and diverse cell lineages. In vertebrate embryos, neural crest cells represent a prime example of a cell type that rapidly transits from an undifferentiated to differentiated state via progressive gene regulatory changes^1^. During the process of central nervous system (CNS) formation, this stem cell population first becomes apparent within the neural folds during neural tube closure by expression of characteristic transcription factors, including *Pax7*, *FoxD3*, *Snai2*, and *Sox10*. After undergoing an epithelial to mesenchymal transition (EMT), neural crest cells exit the neural tube to become migratory cells that home to discrete locations in the periphery and differentiate into diverse cell types, including neurons and glia of the peripheral nervous system, melanocytes of the skin, and cartilage and bone of the face^2^.

While cell lineage tracing experiments have shown that the premigratory neural crest contains multipotent cells that give rise to numerous derivatives^3–5^, there remain arguments in the literature regarding the balance between multipotency and early restriction in neural crest cell fate^6, 7^. Similarly, questions remain regarding the nature, location, or even existence of a neural crest stem cell niche as well as the regulatory mechanisms underlying maintenance of stemness.

Many of the transcription factors comprising the neural crest gene regulatory network are known^1^. Despite this, a systems level view of the transcriptional program and the degree of cell to cell heterogeneity within the dorsal neural tube remains poorly understood due to the challenge of monitoring the regulatory state of individual cells. Moreover, little is known about the expression of pluripotency factors, such as *Oct4*, *Nanog*, or *Klf4*, in the premigratory or migrating neural crest. To tackle this question in the past, conventional in situ hybridization and immunocytochemistry have been routinely used to analyze transcript or protein co-expression. For example, immunolabeling with the neural crest markers FoxD3 and Pax7 in the neural folds reveals some overlapping expression but other cells that express one but not the other marker^8^. Although this might be interpreted as indicating heterogeneity of neural crest precursors, these approaches are qualitative and limited in resolution, specificity and numbers of molecules that can be concurrently identified. While the recent adaptation of RNA-seq to single cells partially compensates for these issues by enabling detailed transcriptional profiling at the individual cell level, a shortfall is that it does not provide spatial information or reliable detection of low copy number genes^9–12^.

To circumvent these problems and enable analysis of subpopulations within the neural crest at the single cell  level in vivo in the avian neural tube, we present an imaging based Spatial Genomic Analysis (SGA) pipeline, which utilizes multiplex analysis of gene expression at  single cell resolution in a manner that preserves spatial information. This has enabled identification of distinct previously uncharacterized neural crest populations in the developing dorsal midbrain. These include a neural crest stem cell niche comprised of cells with combined neural crest and pluripotency markers located around the dorsal midline, surrounded by cells that express neural crest markers without a pluripotent signature. More laterally, there is a neural stem cell domain that may represent a buffer zone of cells that can transit either to neural tube or neural crest domains. Our results provide fundamental insights into the stem cell characteristics of the neural crest in vivo by revealing spatially and transcriptionally distinct subdomains at single cell resolution.

## Results

### Transcriptional profiling by SGA

**Fig. 1:**
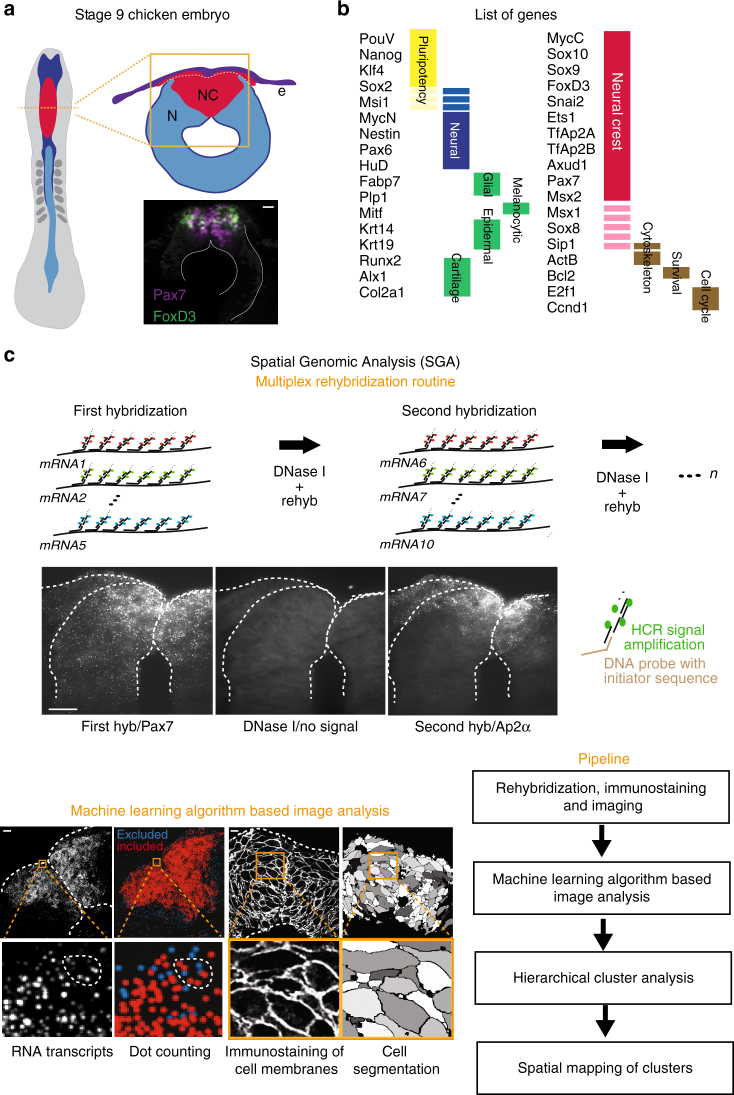
Spatial genomic analysis applied to developing dorsal chick neural tube. **a** Tissue sections were acquired from the midbrain level of stage HH9 chicken embryos. Double immunostaining of the premigratory neural crest using antibodies to Pax7 and FoxD3 reveals a heterogeneous expression pattern. **b** A list of the 35 genes used in the study with their functional roles. **c** The multiplex rehybridization routine was performed as follows: for each round of hybridization, a set of (18–24) DNA probes amplified by using HCR are imaged by using five orthogonal channels. The probes are removed with DNase I treatment between each hybridization step. For the machine learning algorithm based image analysis pipeline, each transcript is visualized as a single diffraction-limited dot in 3D space and filtered from background noise (blue dots are excluded from the analysis). Cell membranes are visualized by immunostaining (using antibodies to E-cadherin and β-catenin). To ensure single cell  accuracy, we developed a computer program to perform cell segmentation based on the stained plasma membranes, and to align individual images between hybridization rounds based on morphological features of the tissue. Number of transcripts is counted with each segmented 3D cell and divided by the cell volume to normalize the data according to cell size variation. Scale bar, 10 μm

In order to study multiplex gene expression in the premigratory neural crest, we used midbrain cross sections from avian dorsal neural tubes (Fig. 1a). SGA was developed by combining sequential single molecule fluorescence in situ hybridization (seqFISH)^13–15^ with hybridization chain reaction (HCR)^16–18^ to amplify signal strength for in vivo tissue applications, thus enabling simultaneous analysis of multiple transcripts on embryonic tissue sections. Transcripts for each gene are visualized with fluorescent DNA probes. For each round, five genes are detected with different fluorophores and imaged by spinning disc confocal microscopy. Probes are then removed by treating with DNase I^19^, and the hybridization is repeated here seven times, enabling identification of 35 genes (Fig. 1b, c). To ensure RNA integrity, the first hybridization set was repeated after the multiplex routine with over 80% signal recovery rate (Supplementary Fig. 1A).

One of the challenges of doing multiple rounds of rehybridization is ascribing transcripts to individual cells. To circumvent this problem for SGA, we developed a machine learning algorithm based image analysis pipeline to segment individual cells and count the transcripts inside them with single molecule accuracy (Fig. 1c, Supplementary Fig. 1, and Supplementary Movie 1). This serial SGA approach enables spatial transcriptional profiling in complex tissue samples at single cell resolution, and can be easily implemented for as many as a hundred genes.

### The dorsal neural tube consists of five subpopulations

To apply SGA to the neural crest system, we designed probes against genes associated with particular cell identities or regulatory states involved in neural crest development, including neural and neural crest markers, pluripotency factors, differentiation markers, and genes associated with cell proliferation or cell death (Fig. 1b). These were then hybridized onto tissue sections through the midbrain region of chick embryos shortly after neural tube closure and at the onset of neural crest migration (7 somite stage, Hamburger and Hamilton HH stage 9, Fig. 1a). Five samples from three different embryos were measured with ~250 cells per sample. Data were pooled to create a single heat map according to the z-scored expression values for each of the 35 genes (Fig. 1).

**Fig. 2:**
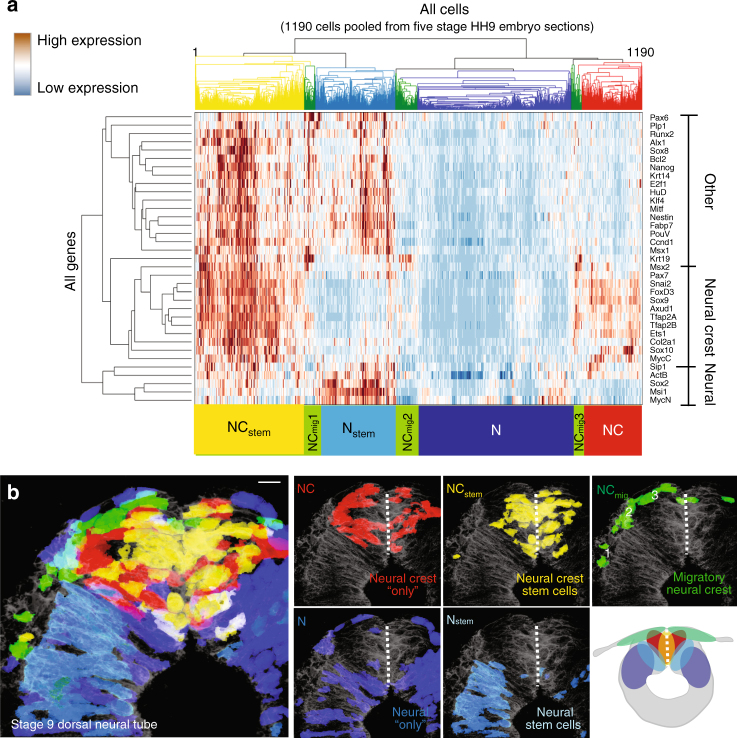
Hierarchical clustering reveals spatially distinct subdomains in the dorsal neural tube. **a** Pooled data from 1190 cells from 5 midbrain cross sections of three embryos reveal two main cell populations: stem cells that express both pluripotency and differentiation markers (yellow and light blue), together with cells without a pluripotent signature (red and blue). These can be further clustered into different subpopulations of neural or neural crest cells. Migrating neural crest cells are in green. Vertical axis shows the relationships between the genes according to similarity in expression pattern. **b** Using SGA, single cells in the heat map can be mapped back to the embryo section to confer spatial information. Five clusters form reproducible spatial patterns in the dorsal neural tube. Neural crest stem cells (NC_stem_) are located around the dorsal midline and surrounded by neural crest cells without expression of pluripotency genes (NC). The migrating neural crest cells (NC_mig_1–3) express *Krt19* and *Msx2*, and form a group of mesenchymal cells outside the neural tube (see also Fig. 3c). Neural cells are found lateral to neural crest cells; neural cells with a pluripotent profile (N_stem_) reflect a border zone between the neural crest and the neural cells (N). Schematic view shows the spatial location of the subdomains in the dorsal neural tube. Scale bar, 10 μm

**Fig. 3:**
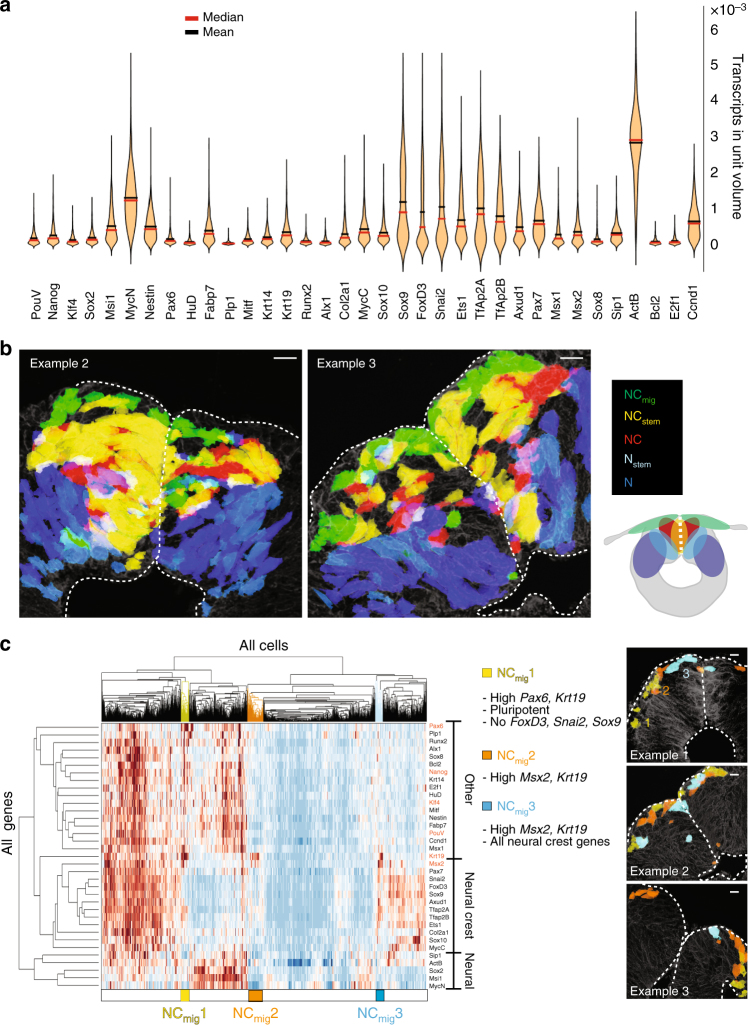
Reproducibility of subclusters between different embryos. **a** Violin plots show modality and gene expression levels for the 35 genes used in the study. Plots are based on Gaussian kernel density estimation and MatLab (R2015a v8.0) default settings for ksdensity command. **b** The five clusters (see heat map in Fig. 2a with pooled data from five samples) form reproducible spatial patterns in the dorsal neural tube. Spatial mapping of the pooled clusters into two additional embryo sections reproduce the pattern seen in Fig. 2b. **c** The early migratory neural crest population (shown in green in Fig. 2) consists of three separate subpopulations (NC_mig_1–3) with spatially distinct locations. All populations express the epidermal marker *Krt19*, whereas the cells that emigrated first and have thus migrated the furthest express pluripotency markers and high levels of the neuroectodermal gene *Pax6*. The following populations (NC_mig_2–3) have high *Msx2* expression. For the subcluster reproducibility analysis, five samples from three different embryos were compared and three representatives were chosen for the images (*n* = 5). Scale bar, 10 μm

Unbiased hierarchical clustering analysis without prior spatial information revealed five distinct cell clusters (Fig. 2a). When these were mapped back to the original tissue sections, they corresponded to five reproducible but previously unrecognized spatial subdomains in the dorsal neural tube (Figs. 2b and 3b). Most cells with a premigratory neural crest signature localized to a heart shaped  region bisected by the midline of the dorsal neural tube: the central-most portion expressed a combination of neural crest, pluripotency, and differentiation markers (NC_stem,_ yellow), whereas the cells just lateral to that expressed neural crest “only” genes (NC, red). Cells with a migratory neural crest signature consisted of three distinct and spatially well defined  subclusters (NC_mig_1–3, green). Interestingly, all NC_mig_ cells express the epidermal marker *Krt19* together with subsets of neural crest markers (Fig. 3c). Lateral to the heart shaped  neural crest cells, we find another population that has high expression of neural markers together with differentiation and pluripotency genes (N_stem,_ light blue). These neural stem cells are bordered by both the neural crest domain, and the more ventral neural (N, blue) cells, which only express neural genes. Accordingly, the two described stem cell populations (yellow and light blue) also have the highest expression of the proliferation markers *Ccnd1* and *E2f1* (Fig. 2a, b). The relative expression levels of each gene are presented as a violin plot in Fig. 3a.

### Distinct pluripotency signature of two stem cell populations

**Fig. 4:**
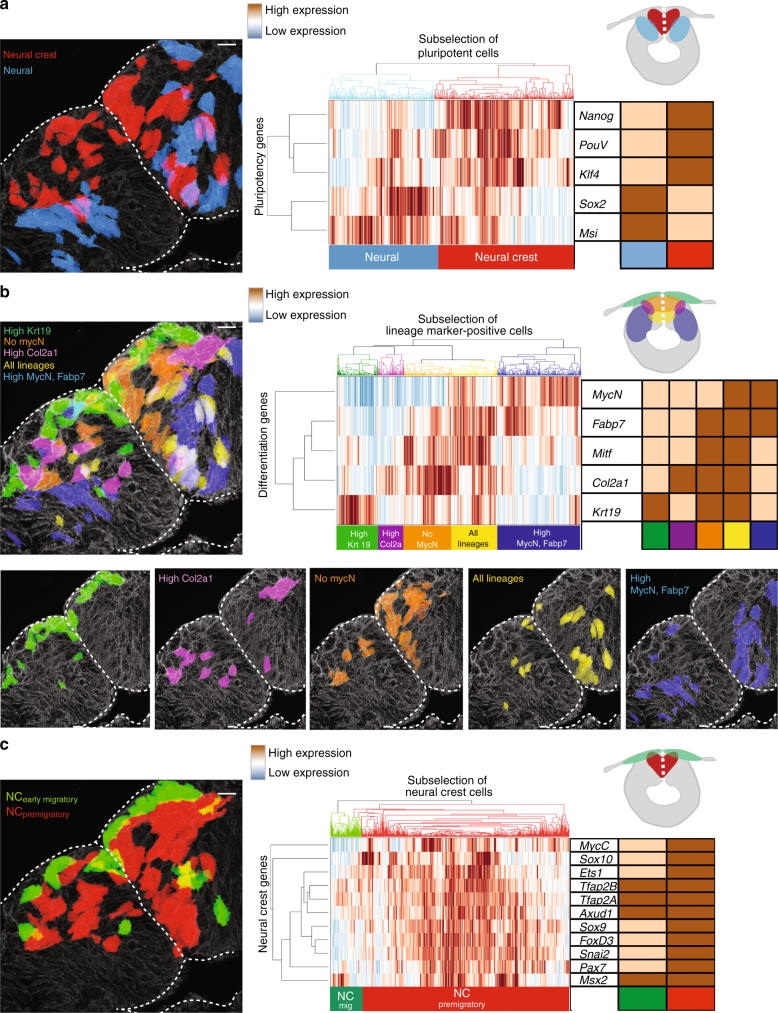
Analysis of functionally distinct genes reveals previously undescribed expression patterns within the dorsal neural tube. For each figure, all 1190 cells were clustered according to a subset of genes. Only the cells expressing the corresponding genes are shown in the clustergrams. A simplified table and schematic representation of the results is included in each panel. **a** Clustering using pluripotency markers separates neural vs. neural crest domains as shown by the hierarchical clustered heat map and the corresponding spatial mapping. Interestingly, these two domains express a different subset of stem cell markers, with neural crest cells predominantly expressing *Nanog*, *PouV*, and *Klf4*. **b** Premigratory neural crest cells also express multiple lineage specific  marker genes. Hierarchical clustering reveals heterogeneous expression profiles reflecting five subclusters. These groups form well defined  and reproducible spatially distinct domains in the dorsal neural tube. The most dorsal cells (including both migratory neural crest and skin ectodermal cells) express high levels of the epidermal marker *Krt19* (green). Another cluster consists of cells mainly expressing the cartilage lineage marker *Col2a1*, located dorsolaterally within the premigratory neural crest domain (purple). The dorsomedial domain expresses markers of all lineages examined except the neural stem cell marker *MycN* (orange). The basomedial domain expresses markers of all lineages including neural, glial, melanocytic, cartilage, and epidermal (yellow). As expected, the cells outside the heart-shaped neural crest domain predominantly express neural and glial genes (blue). **c** Finally, clustering using only neural crest markers reveals distinct expression profiles of migratory vs. premigratory neural crest cells. Premigratory populations generally express all neural crest markers, whereas the migratory cells were chosen based on their expression profile that have a consistent expression of *Tfap2A*, *Tfap2B*, *Axud1*, and *Msx2*. For the subcluster reproducibility analysis, five samples from three different embryos were compared and three representatives were chosen for the images (*n* = 5). Scale bar, 10 μm

Consistent with the previously described multipotent nature of premigratory neural crest cells^4, 5^, our results show that several pluripotency markers indeed are expressed in the dorsal neural tube (Fig. 2). To further explore this finding, we clustered the data according to five pluripotency markers. Interestingly, the results show that different sets of pluripotency genes are associated with neural crest vs. neural populations. Whereas the medially localized neural crest cells express high levels of *Nanog*, *PouV*, and *Klf4*, the more lateral neural stem cells express high levels of *Sox2* and *Msi1* (Fig. 4a and Supplementary Fig. 2A).

### Differentiation marker expression in the dorsal neural tube

Due to our finding that all five differentiation markers are co-expressed with the pluripotency markers, we asked whether the dorsal neural tube can be divided into subregions according to lineage marker expression (Fig. 4b and Supplementary Fig. 2B). Interestingly, cells around the midline of the dorsal neural tube express all lineage markers (yellow) with the distinction that the more dorsally located cells do not express *MycN* (orange). Cells expressing markers for all lineages overlap with pluripotent neural crest cells (Fig. 4b, compare to red cells in Fig. 4a). High *Col2a1*-expressing cells (a marker for cartilage fate) that do not express other lineage markers clustered as an individual group and are located most laterally in the neural crest cell domain. These cells do not overlap with the pluripotent cells (purple cells in Fig. 4b, compare with blue and red in Fig. 4a). Cells with neural and glial features localize ventrolaterally and overlap with the neural stem cells (blue cells in Fig. 4b compare to blue cells in Fig. 4a). In migratory neural crest cells (green), there is high expression of *Krt19* but low expression of other lineage markers. However, some migratory cells also co-express single lineage markers individually (see heat map in Fig. 4b) and some express pluripotency markers (compare green cells in Fig. 4b with red cells in Fig. 4a).

### Neural crest markers in different populations

We also examined the differential expression profile of the premigratory vs. early migrating neural crest cells by parsing the data solely using neural crest genes. Interestingly, the clustering shows that the premigratory population expresses high levels of neural crest markers, including *FoxD3*, *Sox9*, *Snai2 (aka Slug)*, *and MycC*, but that these were downregulated in early migratory cells, which predominantly expressed *Msx2*, *TfAP2α*, *TfAP2β*, and *Axud1* (Fig. 4c and Supplementary Fig. 2C).

### SGA provides insight into gene regulatory networks

**Fig. 5:**
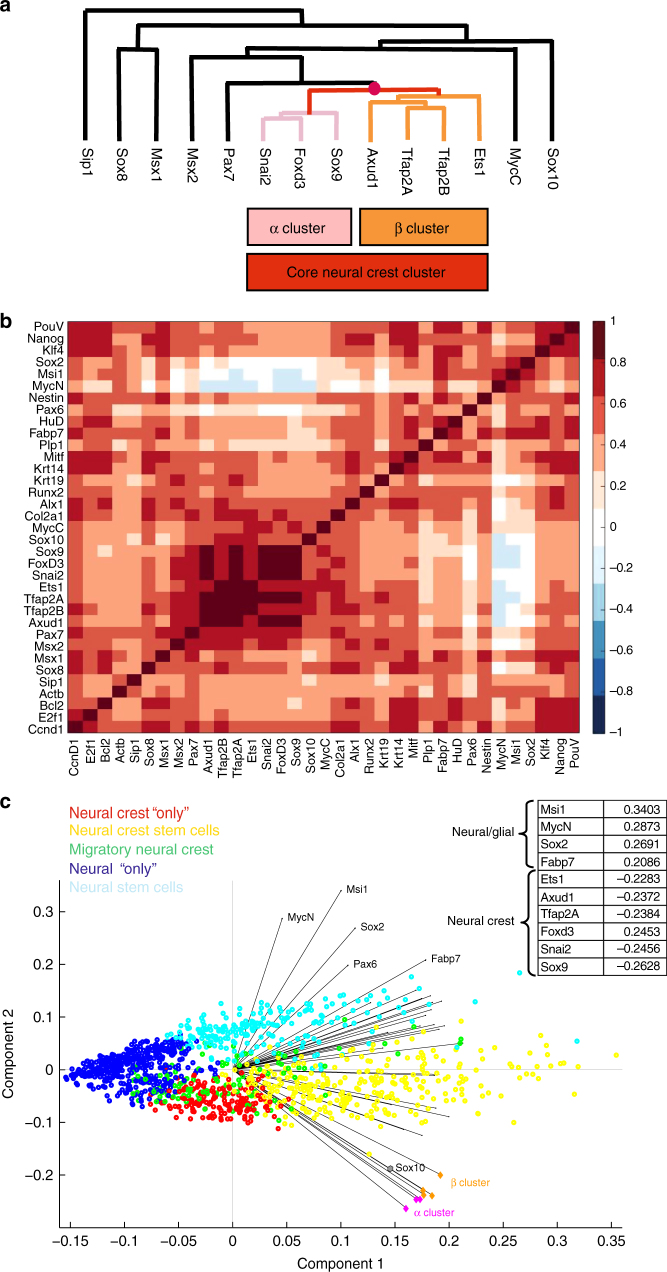
Relationships between neural crest genes based on their co-expression patterns within individual cells. **a** Genes with similar expression profiles are more closely clustered on the dendrogram, suggesting regulatory relationships. Hierarchical clustering analysis of genes reveals a core neural crest cluster of seven genes that are the most likely to be expressed simultaneously in individual cells in the stage HH9 chicken embryo. The core cluster is further subdivided into two subclusters (α and β). However, *Sox10* has a different expression profile compared with the core neural crest genes and other *SoxE* genes. *Sip1*, *Sox8*, and *Msx1* cluster even further away, suggesting a different function from the core neural crest genes. **b** The 35 × 35 correlation matrix of all the genes from all 1190 cells. Red values reflect positive correlation, white means no correlation, and blue values reflect anti-correlation. **c** Plot of the first two components of principal component analysis (PCA). Cells are color coded according to their cluster definition in Fig. 2 (neural crest “only,” neural crest stem cells, migratory neural crest, neural “only,” and neural stem cells). The positions of the cells indicate the relative score of each of the 1190 cells, scaled with respect to the maximum score value and maximum coefficient length. Black vectors indicate contribution of each of the 35 genes from the first two components. Note that the neural crest genes clearly separate from neural genes, and that Sox10 is separate from the core cluster of neural crest genes. Black solid lines around 0 are to be used as a guide for the eye. The greatest spread in genes is found in the principal component two. The 10 genes showing the highest absolute values of variability within the component are shown in the insert that clearly separates the core neural crest from the neural and glial genes

Finally, hierarchical clustering of genes revealed correlations between the expression profiles of neural crest genes in individual neural crest cells, indicating genes that are most likely to be transcribed in the same cells at the same time. For example, individual cells displayed synexpression of two subgroups of neural crest genes (Fig. 5a and Supplementary Fig. 3): α (*Sox9, FoxD3, Snai2*) and β (*Ets1,TfAP2α, TfAP2β, Axud1*) clusters, which together represent a core set of neural crest genes, in accordance with previous reports showing co-operative roles for some of these genes^20–22^. On the other hand, the neural crest marker gene *Sox10* clusters outside this core suggesting a separate role. Similarly, the complete 35 × 35 correlation matrix of all genes shows highest correlation between the core neural crest genes (dark red), and anti-correlation (blue) between neural (*MycN* and *Msi1*) and neural crest genes (Fig. 5b). Furthermore, the first two components of the principal component analysis (PCA) recapitulate these major findings regarding the relationships in gene expression of the 35 genes (Fig. 5c). These first two components represent the majority (2/3) of the data variability. Further components become less significant with an additional increase of less than 5% each. The five different cell populations are well separated as their own groups in principal component space. Only the migratory cells (green dots) overlap with others, predominantly with the pluripotent (yellow) and nonpluripotent (red) neural crest cells. This is in line with the possibility that some of the migrating cells are already more progenitor-like (see heat map in Fig. 2a). The genes of the α and β clusters are next to each other further confirming their similar synexpression profile. Finally, PCA highlights the genes with the most variability including the core neural crest genes as well as the neural stem cell markers *Sox2* and *Msi1* together with neural and glial markers *MycN* and *Fabp7* (Fig. 5c).

## Discussion

By taking advantage of machine learning algorithms for defining cell borders, we have developed a SGA approach for simultaneously and unambiguously imaging multiple diffraction limited  transcripts within single cells with full spatial resolution. This is particularly important when dealing with challenging tightly adhered epithelial tissues such as the dorsal neural tube, where the cells are highly intermingled. Since alignment is based on morphological features detected by a computer program developed for this study, samples can be removed from the microscope allowing us to repeatedly measure multiple thick samples at the same time. By enabling simultaneous analysis of numerous genes in single neural crest cells in vivo, SGA reveals previously unknown position based  differences in gene expression among premigratory and early migrating cranial neural crest cells. This has enabled characterization of a previously unknown pluripotent neural crest stem cell niche in the dorsal neural tube. In addition, this new spatial transcriptional profiling approach provides a useful tool that can be applied to a large number of tissues across a variety of biological systems.

Combining multiplex gene expression with spatial information provides intriguing biological insight about distinct stem cell niches in the dorsal neural tube. Our data identify reproducible regulatory states associated with distinct dorsoventral and mediolateral positions that likely represent different functional zones within a heart shaped  neural crest domain. These snapshots of transcriptional profiles suggest that there is position dependent  gene expression in the dorsal neural tube. Whereas the medial most cells appear to represent a proliferative “mother” neural crest stem cell population, we speculate that these cells give rise to the more progenitor-like cells surrounding them.

The single transcript level sensitivity of our method allows quantitative and multiplexed measurement of low copy number genes, such as pluripotency markers, that previously were largely undetected in the dorsal neural tube due to limitations of conventional methods^23^ (Figs. 2 and 3). Interestingly, we show that the pluripotent expression profile of neural crest stem cells (high *Nanog*, *PouV*, *Klf4*) differs from the neural stem cell signature (high *Sox2* and *Msi1*), suggesting a different regulatory mechanism for these two stem cell populations. This is also in line with previous reports showing that repression of *Sox2* is required for the maintenance of the neural crest fate^24^. High *Sox2* levels may, in this context, rather than reflecting pluripotency, represent a neural stem cell status^25, 26^. Furthermore, our findings are in accordance with a recent report by Buitrago-Delgado and colleagues showing that the neural crest in *Xenopus* embryos retains *Vent2* (frog equivalent to *Nanog*) expression and properties characteristic of the early blastula, suggesting that this may reflect a pan-vertebrate trait^27^.

In contrast to reports on embryonic stem cells in which expression of pluripotency and lineage markers does not overlap^28, 29^, we note that many neural crest stem cells concomitantly express both, suggesting that they are open to many choices and/or have largely open chromatin (Figs. 2 and 4 and Supplementary Fig. 2). Interestingly, while the pluripotent (*Klf4*, *PouV*, *Nanog*-positive) neural crest cells express all five different lineage markers, the expression of neural and glial lineage markers is dominant in neural stem cells (*Msi1*, *Sox2*-positive), suggesting a higher stem cell potential of the neural crest stem cells. Given that the same patterns are observed from embryo to embryo, rather than stochastic jumping between different states^30–33^, our results support the idea that there is a gradual transition as cells move between different regulatory states within the dorsal neural tube. Furthermore, our observation of pluripotency markers in some early migrating neural crest cells is consistent with previous studies showing that even migratory neural crest cells can contribute to multiple fates^4, 5, 34^, and share gene signatures similar to early blastula cells^27^.

In parallel with the neural crest domains, we observed two different regulatory states within the neural population, an apparent neural stem cell population that borders the heart shaped neural crest domain and a neural cell population that is more ventrally localized. Single cell lineage analysis has shown that single neural tube cells can contribute to both the CNS and neural crest^4, 5^, and ablation experiments have shown a contribution from lateral regenerating neural tube cells to the neural crest domain^35^. Thus, the neural stem cells may represent a pool that can contribute to both the neural tube and neural crest.

Finally, simultaneous gene expression analysis of all the commonly used neural crest markers shows that they are not expressed uniformly throughout the population, highlighting the importance of using appropriate markers when analyzing experimental results. Hierarchical clustering separates the premigratory and early migrating neural crest cells into two transcriptionally distinguishable subpopulations solely based on their expression of neural crest markers (Fig. 4c). This provides valuable information regarding the potential roles of these markers. First, the fact that expression levels of *FoxD3*, *Sox9*, *Snai2 (Slug)*, and *MycC* are significantly higher in the premigratory neural crest supports their role in stem cell maintenance, pluripotency, and promotion of EMT^36–41^. Second, the separation of neural crest markers into a “core” neural crest cluster further confirms this diversity and suggests different functional roles for genes that lie within vs. outside of the core. Our data also show that multiplex single cell  analysis can be used to evaluate synexpression groups that may help inform upon gene regulatory network relationships within a given tissue (Fig. 5).

In summary, we have developed a single molecule imaging based SGA pipeline and used it to reveal previously unknown stem cell populations in the dorsal neural tube of developing chick midbrain. Our results provide a valuable link between systems biology and classical developmental biology, revealing quantitative information of the developing neural crest. This pipeline can be easily implemented to address many biological questions in multiple organisms.

## Methods

### Sample preparation

Seven somite stage (Hamburger Hamilton HH stage 9) chicken embryos were fixed in 4% paraformaldehyde (PFA) overnight +4 °C, dehydrated in ethanol overnight (−80 °C), washed 3× with PBS-0.2% Triton, and brought to OCT embedding compound (Tissue-Tek 4583) via a 20% sucrose gradient (3 h on a nutator +4 °C). The embryos were snap frozen in liquid nitrogen and 12–16 μm-thick midbrain cross sections were cut and collected to aminosilane-coated (>98%, Aldrich) #1.5 coverslips.

### SGA protocol

Probes were designed as follows: mRNA-binding sequences were designed as a reverse complement of the coding DNA sequence region of the mRNA transcript with minimum of five base separations between the probes and CG-content being between 40 and 60%. When possible, we used 24 probes per gene with the minimum number being 12 for *Fabp7*. The probes were blasted (NCBI) to assure their specificity and the probes were ordered from Integrated DNA Technologies. We designed DNA probes based on previously described HCR initiator sequences^17^. A 18–20 nt long mRNA-binding sequence and a four base linker sequence were added to the 3′ end of each DNA probe (Supplementary Table 1). Hairpins were ordered from Molecular Probes and we used five orthogonal channels (Cy7, Alexa647, Alexa594, Cy3b, and Alexa488) for single molecule imaging.

### Hybridization

Before starting a multi-day hybridization routine, we tested RNA integrity by using a probe set (*Snai2*, 24 probes) where every other probe was labeled with a different initiator sequence. They were then amplified with two orthogonal channels and the diffraction limited dots showed better than 80% colocalization. After the multiplexed hybridization routine, the first set of genes was repeated at the end of the hybridization rounds. Routinely, a more than 80% recovery of the dots further confirmed the RNA integrity over the entire experiment.

All the hybridizations and imaging were done inside hybridization chambers (Grace Bio-Labs, volume ~ 50 μl) attached onto an aminosilane-coated #1.5 cover glass. OCT-medium embedded embryos were treated and fixed on cover glasses by washing the sample several times with 1% PFA in 1× PBS. The samples were then permeabilized with 0.5% SDS in 1× PBS for 10 min and postfixed with 1% PFA in 1× PBS for 10 min. Background autofluorescence was reduced by treating the samples with 1% NaBH_4_ in 1× PBS for 5 min and washed several times with 2× SSC prior hybridization.

The samples were blocked with 1 μM random 60 nt DNA oligomers in 2× SSC for 1 h and the hybridization was performed in chambers at 100% humidity at 37 °C overnight. The probe concentration was 1 nM per individual probe in 10% high molecular weight dextran sulfate (DS) and 30% formamide (FA) in 2× SSC. Thiomerisal (0.01%) was added to the hybridization solution to prevent fungal and bacterial growth. The next day, the samples were washed with 30% FA in 2× SSC (3×) following a SSC wash (2×).

The fluorescent signal was amplified using HCR with corresponding hairpins in 10% DS/2× SSC solution for 1.5 h at room temperature, followed by washes (3×) with 30% FA 2× SSC and 2× SSC yielding a diffraction limited signal for each transcript. The samples were costained with DAPI during the first hybridization round and the samples were imaged under Pyranose Oxidase enzyme (Sigma P4234)based anti-bleaching buffer. After imaging, the DNA probes and the hairpin polymers were digested with 1 h DNase I treatment (0.5 U / μl^−1^, NEB Biolabs M0303) followed by three times 30% FA 2× SSC (3×) and 2× SSC (3×) washes. The routine was repeated for each additional set of hybridizations.

### Immunostaining

In order to define the cell borders for cell segmentation in the dorsal neural tube, cell membranes were immunostained using two separate antibodies against two membrane proteins (β-catenin and E-cadherin) to achieve uniform and maximal signal strength. To avoid RNase contamination during the hybridization phase, the immunostaining was performed last according to standard protocol. The samples were blocked with 5% BSA in 1× PBS-0.2% Triton and 1% DMSO for 1 h at room temperature and incubated over night +4 °C with antibodies to E-cadherin (610181; BD Biosciences 1:1000) and β-catenin (Abcam ab6301clone15B8, 1:750). The sections were then washed 5× with PBS-0.2% Triton and incubated 3 h at room temperature with the respective secondary antibodies with the same fluorophore (Alexa647 goat anti-mouse IgG2a and Alexa647 goat anti-mouse IgG1, 1:1000) and washed for imaging.

### Imaging

Imaging was performed with Yokogawa CSU-W1 spinning disk confocal unit on an Olympus IX-81 microscope using six orthogonal channels. The dyes and their respective laser excitation lines used were: Cy7 (727 nm), Alexa647 (640 nm), Alexa594 (589 nm), Cy3B (532 nm), and Alexa488 (473 nm) for single molecule imaging (sm) and DAPI (405 nm) for nuclei and fluorescent background imaging. Typical exposure time was 500 ms. The camera was back-illuminated CCD Andor iKon-M 934 BEX2-DD that offered a high quantum efficiency (>90%) in a broad spectral range from ultraviolet (400 nm) to near-infrared (850 nm). Pixel size of the camera was 13 × 13 μm and the sensor array consisted of 1024 × 1024 pixels. Z-axis was imaged with 0.5 μm intervals and voxel size (unit volume) was thus 8.5 × 10^−3^ μm^3^. Olympus 10× UPlanFL N 0.3 NA air objective was used for sample alignment and large field of view fluorescence imaging. Images were captured through an oil immersion objective Olympus 100× UPlanSApo 1.4 NA. The channels are fully orthogonal. In order to achieve this, we have used laser illumination and the dichroic and emission filter sets were carefully chosen. We filtered out the noise from the background by excluding the dots with the weakest intensity due to nonspecific binding of the probes (typically consisting of less than 5% of all the dots). Motorized and computer controlled sample mounting stage ASI MS-2000 was used to move and tile the samples when necessary. Image acquisition was performed with a Micro Manager software (v. 1.4.18). Epifluorescence images were captured with the similar system as described above but without the spinning disc confocal unit.

### Image analysis

We created a machine learning based image analysis pipeline to identify single-cell outlines from 3D image stacks. Briefly, the membrane label stain and autofluorescence background was used to train a random forest classifier^42^ that resulted in prediction maps of the cell interiors (Fig. 1c). Using thresholding on these prediction maps, and performing post filtering based on size, we obtained cell interiors that were used as seeds for a watershed algorithm^43^ implemented in MatLab (R2015a v8.0) program. Watershed algorithm provided 3D cell outlines and cell label matrixes that were used as masks for further analysis. This cell segmentation method created a volumetric space for each cell that was filtered out according to Gaussian distribution providing a representative 3D cell population of the tissue section. The majority of the filtered cells were 75% or more intact (see Supplementary Movie 1). We used the same machine-learning software for transcript detection, and the MatLab program counted the dots in segmented cells. The dots are diffraction limited and even in the case of the highest copy number gene β-actin, the dots were non-overlapping. Cell size and the transcript detection thresholds were filtered to accept cell sizes and transcript intensities that followed the Gaussian distribution. Typically, we filtered out <5% of the cells and transcripts depending on the copy number of the corresponding gene. The absolute copy numbers of transcripts were divided with a cell volume to take into account different cell sizes due to a natural variability and the presence of partial cells in the sections. Image alignments were done in an automated fashion using the MatLab software developed for this purpose and corrected manually when necessary. Code is available at www.singlecellanalysis.org or at https://github.com/CaiGroup/neuralcrest.

### Data analysis

Gene expression heat maps and PCA plots were generated based on the z-scored gene expression data using a MatLab program. Hierarchical clustering analysis was done in two dimensions with a cosine similarity between the pairwise objects in the distance matrix. In order to normalize the natural variation of transcript levels between different embryos, pooled data from separate experiments were z-scored prior to pooling. Subsets of cells and/or genes were picked for further analysis and reclustered accordingly using the same clustering method. Cluster groups were mapped back to the spatial domain and visualized by using our MatLab program.

### Data availability

The authors declare that all data supporting the findings of this study are available within the article and its Supplementary Information files or from the corresponding authors on reasonable request.

## Electronic supplementary material

Supplementary Information

Description of Additional Supplementary Files

Supplementary Movie 1
