# Supplementary material for: Identification of a neural crest stem cell niche by Spatial Genomic Analysis
Source: Nat Commun. 2017 Nov 28;8:1830. doi: 10.1038/s41467-017-01561-w (PMC5705662; doi:10.1038/s41467-017-01561-w)
Supplement: Supplementary file 1 — Supplementary Information [file 41467_2017_1561_MOESM1_ESM.pdf]

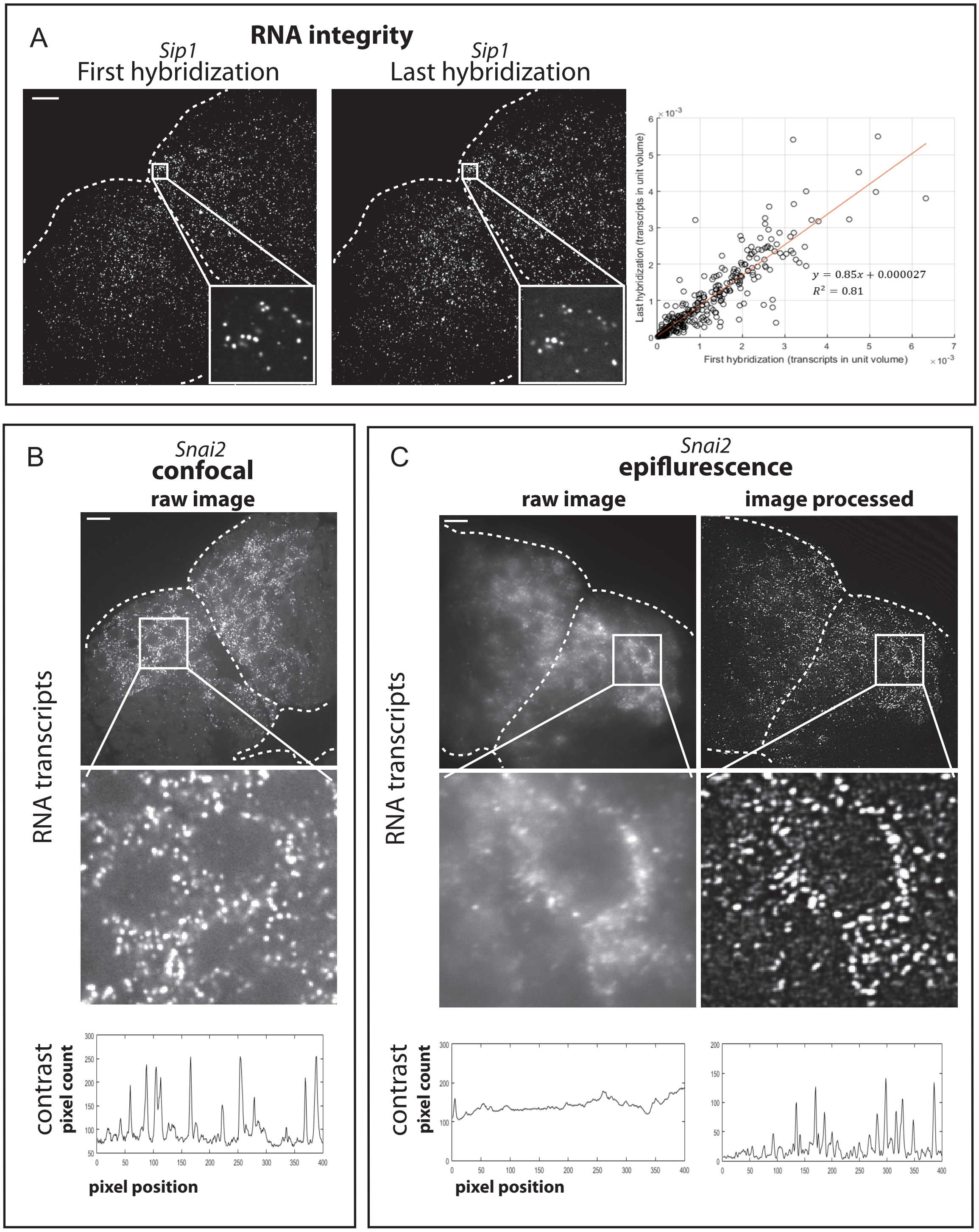

A
RNA integrity
Sip1
First hybridization
Sip1
Last hybridization
Last hybridization (transcripts in unit volume)
First hybridization (transcripts in unit volume)
×10^-3
y = 0.85x + 0.000027
R^2 = 0.81
B
Snai2
confocal
raw image
RNA transcripts
contrast
pixel count
pixel position
C
Snai2
epiflurescence
raw image
image processed
RNA transcripts
contrast
pixel count
pixel position

Supplementary Figure 1

RNA integrity and comparison between imaging methods A) RNA integrity is preserved after multiple rounds of rehybridization (eleven for this image with *Sip1*) as shown by comparison of the raw transcript images as well as the correlation plot (85% of transcripts are preserved). B-C) Spatial Genomic Analysis (SGA) can be performed using an epifluorescent microscope. Comparison of signal to noise ratio between a raw image taken by a spinning disc confocal microscope (A) and an epifluorescent microscope (B) before and after image processing (Laplacian of Gaussian filter) on the Alexa 488 channel that has the highest autofluorescence. The image processed epifluorescent data are comparable to those acquired by confocal microscopy as shown by the pixel values in the graphs. Scale bar 10μm.

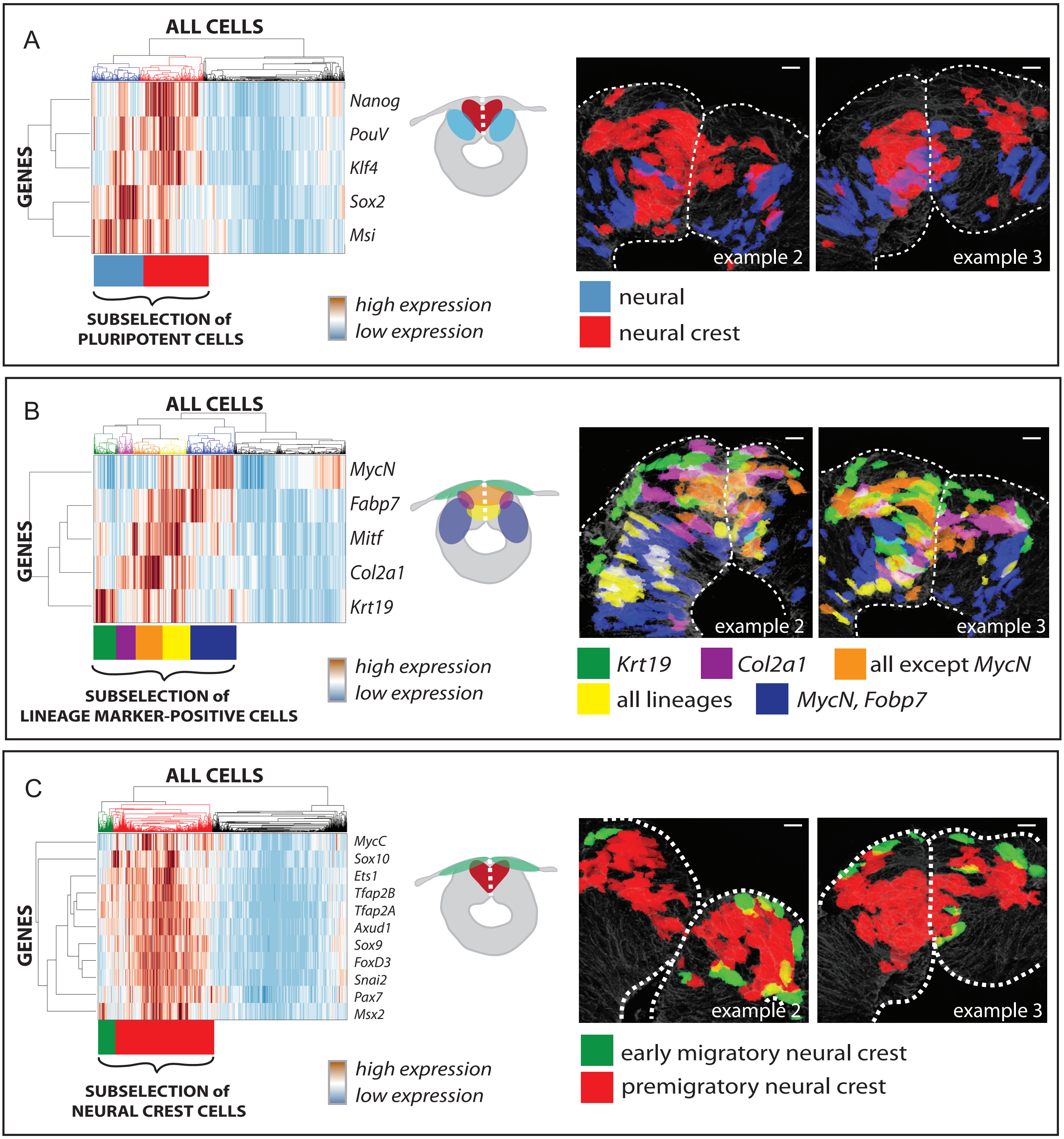
A
ALL CELLS
GENES
Nanog
PouV
Klf4
Sox2
Msi
SUBSELECTION of
PLURIPOTENT CELLS
high expression
low expression
example 2
example 3
neural
neural crest
B
ALL CELLS
GENES
MycN
Fabp7
Mitf
Col2a1
Krt19
SUBSELECTION of
LINEAGE MARKER-POSITIVE CELLS
high expression
low expression
example 2
example 3
Krt19
Col2a1
all except MycN
all lineages
MycN, Fobp7
C
ALL CELLS
GENES
MycC
Sox10
Ets1
Tfap2B
Tfap2A
Axud1
Sox9
FoxD3
Snai2
Pax7
Msx2
SUBSELECTION of
NEURAL CREST CELLS
high expression
low expression
example 2
example 3
early migratory neural crest
premigratory neural crest

Supplementary Figure 2

Reproducibility of the subclusters For each figure, all 1190 cells were clustered according to a subset of genes. A) The cells expressing pluripotency markers were selected for Figure 4A. Examples 2 and 3 show a similar spatial distribution of neural and neural crest stem cell populations as seen in Figure 4A. B) The cells expressing lineage specific markers were selected for Figure 4B. Examples 2 and 3 show a similar spatial distribution of the cell populations as seen in Figure 4A with the Collagen 2a1 expressing cells found lateral to the "all marker" expression zones, and the migrating cells are defined by expression of *Krt19.* C) The cells expressing neural crest markers were selected for Figure 4C. Examples 2 and 3 show a similar spatial distribution of migratory and premigratory neural crest cell populations as seen in Figure 4C. For the subcluster reproducibility analysis, five samples from three different embryos were compared and three representatives were chosen for the images (n=5). Scale bar 10μm.

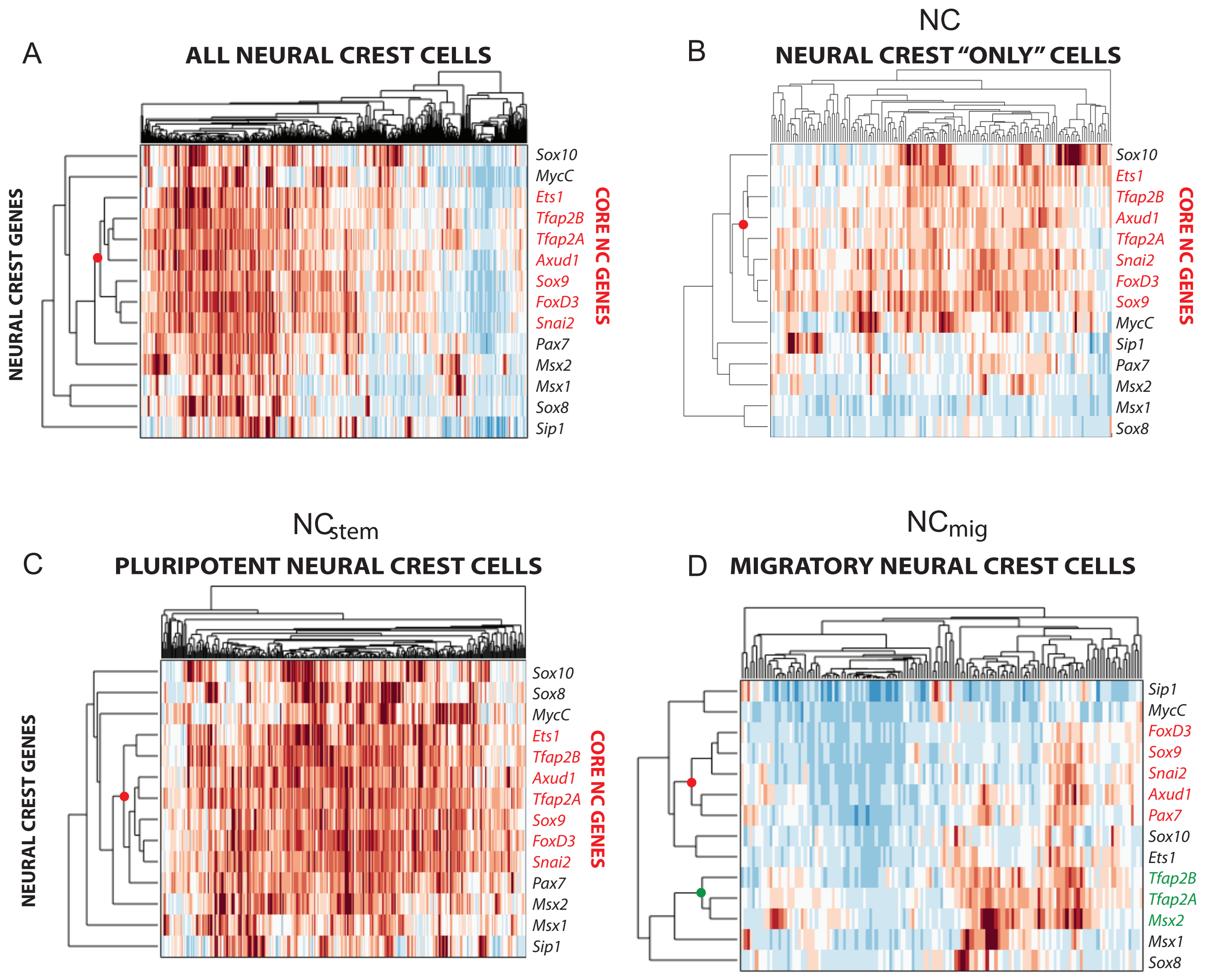
A
ALL NEURAL CREST CELLS
NEURAL CREST GENES
Sox10
MycC
Ets1
Tfap2B
Tfap2A
Axud1
Sox9
FoxD3
Snai2
Pax7
Msx2
Msx1
Sox8
Sip1
CORE NC GENES
NC
B
NEURAL CREST "ONLY" CELLS
Sox10
Ets1
Tfap2B
Axud1
Tfap2A
Snai2
FoxD3
Sox9
MycC
Sip1
Pax7
Msx2
Msx1
Sox8
CORE NC GENES
NCstem
C
PLURIPOTENT NEURAL CREST CELLS
NEURAL CREST GENES
Sox10
Sox8
MycC
Ets1
Tfap2B
Axud1
Tfap2A
Sox9
FoxD3
Snai2
Pax7
Msx2
Msx1
Sip1
CORE NC GENES
NCmig
D
MIGRATORY NEURAL CREST CELLS
Sip1
MycC
FoxD3
Sox9
Snai2
Axud1
Pax7
Sox10
Ets1
Tfap2B
Tfap2A
Msx2
Msx1
Sox8

Supplementary Figure 3

Identification of core neural crest genes in different subpopulations Genes with similar expression profiles are more closely clustered in the hierarchical tree. A) The dendrogram in Figure 5 is shown here together with the original heatmap in order to see how the expression of different genes is distributed among the neural crest cell populations. As shown separately in Panels B-D, this clustergram consists of all three different neural crest populations. B-C) The hierarchical clustering of neural crest genes in the NC population and in the NC_{stem} population, respectively, are similar to each other and to the dendrogram seen with the pooled data in A. Both populations show the highest similarity among the seven core NC genes (shown in red) and the α cluster (*Sox9, FoxD3* and *Snai2*). D) Migratory neural crest cells have a distinct expression profile and neural crest genes cluster in a slightly different manner in these cells: while the α cluster is the same as in premigratory cells, but now groups with only five of the core genes (shown in red); *TfAP2α, TfAP2β,* and *Msx2* form a separate cluster (shown in green).

Hyb 1

Foxd3 B1 1	gtctggccggacatatcgTATAGCATTCTTTCTTGAGGAGGGCAGCAAACGGAAGAG
Foxd3 B1 2	accacgtcgatatccacgTATAGCATTCTTTCTTGAGGAGGGCAGCAAACGGAAGAG
Foxd3 B1 3	ctcgcgggggatcttcacTATAGCATTCTTTCTTGAGGAGGGCAGCAAACGGAAGAG
Foxd3 B1 4	gaagccctgcatcatgagTATAGCATTCTTTCTTGAGGAGGGCAGCAAACGGAAGAG
Foxd3 B1 5	ggatgtaggggtactgcaTATAGCATTCTTTCTTGAGGAGGGCAGCAAACGGAAGAG
Foxd3 B1 6	ttgaaggctttgcggctcTATAGCATTCTTTCTTGAGGAGGGCAGCAAACGGAAGAG
Foxd3 B1 7	gggctcggatttcacgatTATAGCATTCTTTCTTGAGGAGGGCAGCAAACGGAAGAG
Foxd3 B1 8	ctgaacgaggggcggctgTATAGCATTCTTTCTTGAGGAGGGCAGCAAACGGAAGAG
Foxd3 B1 9	aggaaagtctgcgcgctgTATAGCATTCTTTCTTGAGGAGGGCAGCAAACGGAAGAG
Foxd3 B1 10	cgactgcacggtgacgggTATAGCATTCTTTCTTGAGGAGGGCAGCAAACGGAAGAG
Foxd3 B1 11	cggtggtcctggctaacgTATAGCATTCTTTCTTGAGGAGGGCAGCAAACGGAAGAG
Foxd3 B1 12	ctgaggatgggcgcgatgTATAGCATTCTTTCTTGAGGAGGGCAGCAAACGGAAGAG
Foxd3 B1 13	gcgatgatgttggtaggcTATAGCATTCTTTCTTGAGGAGGGCAGCAAACGGAAGAG
Sox9 B2 1	aaggggtctaggagattcatAAAAAGCTCAGTCCATCCTCGTAAATCCTCATCAATCATC
Sox9 B2 2	ctgttcttctgtcattttcaAAAAAGCTCAGTCCATCCTCGTAAATCCTCATCAATCATC
Sox9 B2 3	cggagtcatccgacatggtgAAAAAGCTCAGTCCATCCTCGTAAATCCTCATCAATCATC
Sox9 B2 4	gaaggtgttctcttgaggtcAAAAAGCTCAGTCCATCCTCGTAAATCCTCATCAATCATC
Sox9 B2 5	atgcacacggggaatttgtcAAAAAGCTCAGTCCATCCTCGTAAATCCTCATCAATCATC
Sox9 B2 6	tcacgtggggtttgttcttgAAAAAGCTCAGTCCATCCTCGTAAATCCTCATCAATCATC
Sox9 B2 7	ttgtgcagatgcgggtactgAAAAAGCTCAGTCCATCCTCGTAAATCCTCATCAATCATC
Sox9 B2 8	ttctcgctctcattcagcagAAAAAGCTCAGTCCATCCTCGTAAATCCTCATCAATCATC
Sox9 B2 9	aagatggcgttgggggagatAAAAAGCTCAGTCCATCCTCGTAAATCCTCATCAATCATC
Sox9 B2 10	ctgatgctggaggatgactgAAAAAGCTCAGTCCATCCTCGTAAATCCTCATCAATCATC
Sox9 B2 11	cgtctcggaaatcgatgtggAAAAAGCTCAGTCCATCCTCGTAAATCCTCATCAATCATC
Sox9 B2 12	gagatgacgtcgctgctgagAAAAAGCTCAGTCCATCCTCGTAAATCCTCATCAATCATC
Sox9 B2 13	gacgtcgaaggtttcgatgtAAAAAGCTCAGTCCATCCTCGTAAATCCTCATCAATCATC
Sox9 B2 14	gcaggtattggtcgaactcgAAAAAGCTCAGTCCATCCTCGTAAATCCTCATCAATCATC
Sox9 B2 15	tagtgctgcaggttgaaggaAAAAAGCTCAGTCCATCCTCGTAAATCCTCATCAATCATC
Sox9 B2 16	cggtgtaatcgtactgcgagAAAAAGCTCAGTCCATCCTCGTAAATCCTCATCAATCATC
Sox9 B2 17	tagtaggagccggagttctgAAAAAGCTCAGTCCATCCTCGTAAATCCTCATCAATCATC
Sox9 B2 18	aggtgaaggtggagtagaggAAAAAGCTCAGTCCATCCTCGTAAATCCTCATCAATCATC
Sox9 B2 19	ctgcgatggggggtgtacatgAAAAAGCTCAGTCCATCCTCGTAAATCCTCATCAATCATC
Sox9 B2 20	tgtgggtctgcgggatagagAAAAAGCTCAGTCCATCCTCGTAAATCCTCATCAATCATC
Bcl2 B3 1	ttgtcgtagcctcttctcTAAAAAAGTCTAATCCGTCCCTGCCTCTATATCTCCACTC
Bcl2 B3 2	tgtacttcagcactatctTAAAAAAGTCTAATCCGTCCCTGCCTCTATATCTCCACTC
Bcl2 B3 3	ccgctgcgagagtttataTAAAAAAGTCTAATCCGTCCCTGCCTCTATATCTCCACTC
Bcl2 B3 4	tcactagcagcagccgagTAAAAAAGTCTAATCCGTCCCTGCCTCTATATCTCCACTC
Bcl2 B3 5	tagcggcgcgagaactcgTAAAAAAGTCTAATCCGTCCCTGCCTCTATATCTCCACTC
Bcl2 B3 6	gacatctgggcgaagtccTAAAAAAGTCTAATCCGTCCCTGCCTCTATATCTCCACTC
Bcl2 B3 7	cgtgaagggcgtcaggtgTAAAAAAGTCTAATCCGTCCCTGCCTCTATATCTCCACTC
Bcl2 B3 8	tccaccacggccacgaagTAAAAAAGTCTAATCCGTCCCTGCCTCTATATCTCCACTC
Bcl2 B3 9	ttgaccccatcacggaagTAAAAAAGTCTAATCCGTCCCTGCCTCTATATCTCCACTC
Bcl2 B3 10	aagaaggcgacgatccggTAAAAAAGTCTAATCCGTCCCTGCCTCTATATCTCCACTC
Bcl2 B3 11	tcgacgcacatcacgccgTAAAAAAGTCTAATCCGTCCCTGCCTCTATATCTCCACTC
Bcl2 B3 12	gcgacatctcccggttgaTAAAAAAGTCTAATCCGTCCCTGCCTCTATATCTCCACTC
Bcl2 B3 13	ggtggcaatgttgtccacTAAAAAAGTCTAATCCGTCCCTGCCTCTATATCTCCACTC
Bcl2 B3 14	cggttcaggtactcggtcTAAAAAAGTCTAATCCGTCCCTGCCTCTATATCTCCACTC
Bcl2 B3 15	aaggcctcatactgttgcTAAAAAAGTCTAATCCGTCCCTGCCTCTATATCTCCACTC
Bcl2 B3 16	ggtcttcagagagatccaTAAAAAAGTCTAATCCGTCCCTGCCTCTATATCTCCACTC
Bcl2 B3 17	tgcaagctcccaccagaaTAAAAAAGTCTAATCCGTCCCTGCCTCTATATCTCCACTC
Bcl2 B3 18	tgtccaagataagcgccaTAAAAAAGTCTAATCCGTCCCTGCCTCTATATCTCCACTC
Sip1 B4 1	gcttcggggtaaataatggcATTTCACATTTACAGACCTCAACCTACCTCCAACTCTCAC
Sip1 B4 2	acgagaagttctcctcattcATTTCACATTTACAGACCTCAACCTACCTCCAACTCTCAC
Sip1 B4 3	gtaggcaaacgtgtagctacATTTCACATTTACAGACCTCAACCTACCTCCAACTCTCAC
Sip1 B4 4	gcatttgaacttgcgattgcATTTCACATTTACAGACCTCAACCTACCTCCAACTCTCAC
Sip1 B4 5	aaccgatacacttcttgctgATTTCACATTTACAGACCTCAACCTACCTCCAACTCTCAC
Sip1 B4 6	tctcattctgccatttacagATTTCACATTTACAGACCTCAACCTACCTCCAACTCTCAC
Sip1 B4 7	ttaggagaagaacccgtcttATTTCACATTTACAGACCTCAACCTACCTCCAACTCTCAC

Sip1 B4 8	tagttgcactaaacccatgtATTTCACATTTACAGACCTCAACCTACCTCCAACTCTCAC
Sip1 B4 9	aagccaagtaatggtgcttcATTTCACATTTACAGACCTCAACCTACCTCCAACTCTCAC
Sip1 B4 10	accttcaacttggagatctcATTTCACATTTACAGACCTCAACCTACCTCCAACTCTCAC
Sip1 B4 11	tggcaccattatgacttactATTTCACATTTACAGACCTCAACCTACCTCCAACTCTCAC
Sip1 B4 12	caagctttggcttcattgacATTTCACATTTACAGACCTCAACCTACCTCCAACTCTCAC
Sip1 B4 13	ttatcttcagttaccaggtcATTTCACATTTACAGACCTCAACCTACCTCCAACTCTCAC
Sip1 B4 14	ggagtggatacgttatggttATTTCACATTTACAGACCTCAACCTACCTCCAACTCTCAC
Sip1 B4 15	taacgctcatgctgatgcaaATTTCACATTTACAGACCTCAACCTACCTCCAACTCTCAC
Sip1 B4 16	atggtgacctggaacttgagATTTCACATTTACAGACCTCAACCTACCTCCAACTCTCAC
Sip1 B4 17	tatagagtccacaggctttaATTTCACATTTACAGACCTCAACCTACCTCCAACTCTCAC
Sip1 B4 18	acactgttatggagttcagcATTTCACATTTACAGACCTCAACCTACCTCCAACTCTCAC
Sip1 B4 19	tagtgcttttatccaggttaATTTCACATTTACAGACCTCAACCTACCTCCAACTCTCAC
Sip1 B4 20	aaggtttggcactgaatgggATTTCACATTTACAGACCTCAACCTACCTCCAACTCTCAC
Sip1 B4 21	ctcttctggaatgtcttgtcATTTCACATTTACAGACCTCAACCTACCTCCAACTCTCAC
Sip1 B4 22	aggcgtgaatgctcaatcagATTTCACATTTACAGACCTCAACCTACCTCCAACTCTCAC
Sip1 B4 23	ctatcgtccattgagtgatcATTTCACATTTACAGACCTCAACCTACCTCCAACTCTCAC
Sip1 B4 24	gtcttcttcgtgatctgattATTTCACATTTACAGACCTCAACCTACCTCCAACTCTCAC
Actb B5 1	cgcagcaatatcatcatccATTTCACTTCATATCACTCACTCCCAATCTCTATCTACCC
Actb B5 2	ataccggagccattgtcaaATTTCACTTCATATCACTCACTCCCAATCTCTATCTACCC
Actb B5 3	cccacgatagatgggaacaATTTCACTTCATATCACTCACTCCCAATCTCTATCTACCC
Actb B5 4	ggctactctcagctcattgATTTCACTTCATATCACTCACTCCCAATCTCTATCTACCC
Actb B5 5	ctgtgagcagcacagggtgATTTCACTTCATATCACTCACTCCCAATCTCTATCTACCC
Actb B5 6	ttggctttggggttcagggATTTCACTTCATATCACTCACTCCCAATCTCTATCTACCC
Actb B5 7	acagcctggatggctacatATTTCACTTCATATCACTCACTCCCAATCTCTATCTACCC
Actb B5 8	agagtccatcacaataccaATTTCACTTCATATCACTCACTCCCAATCTCTATCTACCC
Actb B5 9	cacagtgtgggtaacaccaATTTCACTTCATATCACTCACTCCCAATCTCTATCTACCC
Actb B5 10	gagggcgtagccttcatagATTTCACTTCATATCACTCACTCCCAATCTCTATCTACCC
Actb B5 11	agatccagacggaggatggATTTCACTTCATATCACTCACTCCCAATCTCTATCTACCC
Actb B5 12	gtcacgcacaatttctctcATTTCACTTCATATCACTCACTCCCAATCTCTATCTACCC
Actb B5 13	ccatctcctgctcgaaatcATTTCACTTCATATCACTCACTCCCAATCTCTATCTACCC
Actb B5 14	ctccagggaagagctagagATTTCACTTCATATCACTCACTCCCAATCTCTATCTACCC
Actb B5 15	tgaccatcagggagttcatATTTCACTTCATATCACTCACTCCCAATCTCTATCTACCC
Actb B5 16	aacctctcattgccaatggATTTCACTTCATATCACTCACTCCCAATCTCTATCTACCC
Actb B5 17	cactgtgttggcatacagaATTTCACTTCATATCACTCACTCCCAATCTCTATCTACCC
Actb B5 18	ctgcatcctgtcagcaatgATTTCACTTCATATCACTCACTCCCAATCTCTATCTACCC
Actb B5 19	gacagggaggccaggatagATTTCACTTCATATCACTCACTCCCAATCTCTATCTACCC
Actb B5 20	gtccggattcatcgtactcATTTCACTTCATATCACTCACTCCCAATCTCTATCTACCC

Hyb 2

MycC B1 1	taatcgtagttcttgctgggTATAGCATTCTTTCTTGAGGAGGGCAGCAAACGGGAAGAG
MycC B1 2	gaagtagaagtagggctgcaTATAGCATTCTTTCTTGAGGAGGGCAGCAAACGGGAAGAG
MycC B1 3	gctcaaacttcttccagatgTATAGCATTCTTTCTTGAGGAGGGCAGCAAACGGGAAGAG
MycC B1 4	cagatgaagctctggttgacTATAGCATTCTTTCTTGAGGAGGGCAGCAAACGGGAAGAG
MycC B1 5	ttgacgaaggattcgtcgtcTATAGCATTCTTTCTTGAGGAGGGCAGCAAACGGGAAGAG
MycC B1 6	atgcagtcctggatgatgatTATAGCATTCTTTCTTGAGGAGGGCAGCAAACGGGAAGAG
MycC B1 7	ctgagcgggtaggggaagacTATAGCATTCTTTCTTGAGGAGGGCAGCAAACGGGAAGAG
MycC B1 8	tcttgttcttcttccgagtcTATAGCATTCTTTCTTGAGGAGGGCAGCAAACGGGAAGAG
MycC B1 9	gacatcgatttcctcatcttTATAGCATTCTTTCTTGAGGAGGGCAGCAAACGGGAAGAG
MycC B1 10	cgttcgcttcagctaatgtaTATAGCATTCTTTCTTGAGGAGGGCAGCAAACGGGAAGAG
MycC B1 11	gactctgtgctggattcagaTATAGCATTCTTTCTTGAGGAGGGCAGCAAACGGGAAGAG
MycC B1 12	tggatgttgacgtgacaccgTATAGCATTCTTTCTTGAGGAGGGCAGCAAACGGGAAGAG
MycC B1 13	tgtccaactttagcctcttgTATAGCATTCTTTCTTGAGGAGGGCAGCAAACGGGAAGAG
MycC B1 14	gactggagcattttcggttgTATAGCATTCTTTCTTGAGGAGGGCAGCAAACGGGAAGAG
MycC B1 15	tcttgtcgttctcctctgagTATAGCATTCTTTCTTGAGGAGGGCAGCAAACGGGAAGAG
MycC B1 16	tccaagacgttgtgcgttcgTATAGCATTCTTTCTTGAGGAGGGCAGCAAACGGGAAGAG
MycC B1 17	aactcagcttcagctcattcTATAGCATTCTTTCTTGAGGAGGGCAGCAAACGGGAAGAG
MycC B1 18	atctggtcacgcagggcaaaTATAGCATTCTTTCTTGAGGAGGGCAGCAAACGGGAAGAG
MycC B1 19	ttcaggatgacaaccttgggTATAGCATTCTTTCTTGAGGAGGGCAGCAAACGGGAAGAG
MycC B1 20	cagaacgtactccgtggcttTATAGCATTCTTTCTTGAGGAGGGCAGCAAACGGGAAGAG

MycC B1 21	tgtgctcgtccgattggataTATAGCATTCTTTCTTGAGGAGGGCAGCAAACGGGAAGAG
MycC B1 22	tgctctttctctgcgattagTATAGCATTCTTTCTTGAGGAGGGCAGCAAACGGGAAGAG
MycC B1 23	tgtgtttcaactgttctctcTATAGCATTCTTTCTTGAGGAGGGCAGCAAACGGGAAGAG
MycC B1 24	tatgcacgagagttccttagTATAGCATTCTTTCTTGAGGAGGGCAGCAAACGGGAAGAG
Msx2 B2 1	ttcgctttggaaggagaagcAAAAAGCTCAGTCCATCCTCGTAAATCCTCATCAATCATC
Msx2 B2 2	tcctcgtcggaggagaaaacAAAAAGCTCAGTCCATCCTCGTAAATCCTCATCAATCATC
Msx2 B2 3	gacaccttgactttgtggtgAAAAAGCTCAGTCCATCCTCGTAAATCCTCATCAATCATC
Msx2 B2 4	gaaggtttttgtaagagcccAAAAAGCTCAGTCCATCCTCGTAAATCCTCATCAATCATC
Msx2 B2 5	gttctccgatttgaccgaagAAAAAGCTCAGTCCATCCTCGTAAATCCTCATCAATCATC
Msx2 B2 6	tcttgtgcttcctcagagtgAAAAAGCTCAGTCCATCCTCGTAAATCCTCATCAATCATC
Msx2 B2 7	gggaagtagtgaatggggtgAAAAAGCTCAGTCCATCCTCGTAAATCCTCATCAATCATC
Msx2 B2 8	tgagggagctggagaattcgAAAAAGCTCAGTCCATCCTCGTAAATCCTCATCAATCATC
Msx2 B2 9	ttgacctgggtctctgtgagAAAAAGCTCAGTCCATCCTCGTAAATCCTCATCAATCATC
Msx2 B2 10	cctccgattttggaaccagaAAAAAGCTCAGTCCATCCTCGTAAATCCTCATCAATCATC
Msx2 B2 11	attttgagcttctctagctcAAAAAGCTCAGTCCATCCTCGTAAATCCTCATCAATCATC
Msx2 B2 12	cgacggcaacattggcttcgAAAAAGCTCAGTCCATCCTCGTAAATCCTCATCAATCATC
Msx2 B2 13	agttgatggggaaagggaggAAAAAGCTCAGTCCATCCTCGTAAATCCTCATCAATCATC
Msx2 B2 14	taggatgttccgtacagtgaAAAAAGCTCAGTCCATCCTCGTAAATCCTCATCAATCATC
Msx2 B2 15	ataggaagcacaggtctgtgAAAAAGCTCAGTCCATCCTCGTAAATCCTCATCAATCATC
Msx2 B2 16	gagtagcatagagtccaacgAAAAAGCTCAGTCCATCCTCGTAAATCCTCATCAATCATC
Msx2 B2 17	tggtacatgctatatccgacAAAAAGCTCAGTCCATCCTCGTAAATCCTCATCAATCATC
Msi1 B3 1	aagttggttaaagtggcggcTAAAAAAGTCTAATCCGTCCCTGCCTCTATATCTCCACTC
Msi1 B3 2	ggggatgcagagagaagagaTAAAAAAGTCTAATCCGTCCCTGCCTCTATATCTCCACTC
Msi1 B3 3	tggtccatgaacgtgacgaaTAAAAAAGTCTAATCCGTCCCTGCCTCTATATCTCCACTC
Msi1 B3 4	tggattgggccagaaccttgTAAAAAAGTCTAATCCGTCCCTGCCTCTATATCTCCACTC
Msi1 B3 5	gtcttggagtccagttcatgTAAAAAAGTCTAATCCGTCCCTGCCTCTATATCTCCACTC
Msi1 B3 6	gaacgctaccttcgggtcaaTAAAAAAGTCTAATCCGTCCCTGCCTCTATATCTCCACTC
Msi1 B3 7	atcttcttcgttcgggtcacTAAAAAAGTCTAATCCGTCCCTGCCTCTATATCTCCACTC
Msi1 B3 8	cactgacagaccacccacaaTAAAAAAGTCTAATCCGTCCCTGCCTCTATATCTCCACTC
Msi1 B3 9	tttcacatcctccacagtagTAAAAAAGTCTAATCCGTCCCTGCCTCTATATCTCCACTC
Msi1 B3 10	tcccaaactgctcgaaatacTAAAAAAGTCTAATCCGTCCCTGCCTCTATATCTCCACTC
Msi1 B3 11	acgccactcaagagacttacTAAAAAAGTCTAATCCGTCCCTGCCTCTATATCTCCACTC
Msi1 B3 12	ccagaagcaacagctttgatTAAAAAAGTCTAATCCGTCCCTGCCTCTATATCTCCACTC
Msi1 B3 13	gcaaaacttcttcctgcttgTAAAAAAGTCTAATCCGTCCCTGCCTCTATATCTCCACTC
Msi1 B3 14	gtgacaaatccaaaccctgaTAAAAAAGTCTAATCCGTCCCTGCCTCTATATCTCCACTC
Msi1 B3 15	agcttggaagcctggataacTAAAAAAGTCTAATCCGTCCCTGCCTCTATATCTCCACTC
Msi1 B3 16	tgtaacttcgactcgcgtagTAAAAAAGTCTAATCCGTCCCTGCCTCTATATCTCCACTC
Msi1 B3 17	tctacacggaactcagggaaTAAAAAAGTCTAATCCGTCCCTGCCTCTATATCTCCACTC
Msi1 B3 18	attggtccatacgcagtgagTAAAAAAGTCTAATCCGTCCCTGCCTCTATATCTCCACTC
Msi1 B3 19	agggagggaagtggcagttaTAAAAAAGTCTAATCCGTCCCTGCCTCTATATCTCCACTC
Msi1 B3 20	cagaaatccaccggtgcgacTAAAAAAGTCTAATCCGTCCCTGCCTCTATATCTCCACTC
Msi1 B3 21	tccataaagctctgccatcgTAAAAAAGTCTAATCCGTCCCTGCCTCTATATCTCCACTC
Msi1 B3 22	agcgctgatgtaactgctaaTAAAAAAGTCTAATCCGTCCCTGCCTCTATATCTCCACTC
Msi1 B3 23	ctgttgcaatcaagggacccTAAAAAAGTCTAATCCGTCCCTGCCTCTATATCTCCACTC
Msi1 B3 24	cagtgatacccattggtaaaTAAAAAAGTCTAATCCGTCCCTGCCTCTATATCTCCACTC
Klf4 B4 1	tgaactcctcggtttcacgATTTCACATTTACAGACCTCAACCTACCTCCAACTCTCAC
Klf4 B4 2	tgcatgagggagttggagaATTTCACATTTACAGACCTCAACCTACCTCCAACTCTCAC
Klf4 B4 3	caggttgaagggagcggcgATTTCACATTTACAGACCTCAACCTACCTCCAACTCTCAC
Klf4 B4 4	catcagctcggccacgaagATTTCACATTTACAGACCTCAACCTACCTCCAACTCTCAC
Klf4 B4 5	ttgattttggggcacatccATTTCACATTTACAGACCTCAACCTACCTCCAACTCTCAC
Klf4 B4 6	tggagaagtcgtgttgcggATTTCACATTTACAGACCTCAACCTACCTCCAACTCTCAC
Klf4 B4 7	atcagctcttggtactgcaATTTCACATTTACAGACCTCAACCTACCTCCAACTCTCAC
Klf4 B4 8	ttctttttcgaggccatgaATTTCACATTTACAGACCTCAACCTACCTCCAACTCTCAC
Klf4 B4 9	cgtagtcacaggtgtgagtATTTCACATTTACAGACCTCAACCTACCTCCAACTCTCAC
Klf4 B4 10	aggtgtgccttgagatgagATTTCACATTTACAGACCTCAACCTACCTCCAACTCTCAC
Klf4 B4 11	ttcccagtcacagtgataaATTTCACATTTACAGACCTCAACCTACCTCCAACTCTCAC
Klf4 B4 12	gatcgggcaaacttccatcATTTCACATTTACAGACCTCAACCTACCTCCAACTCTCAC
Klf4 B4 13	ccgtgtgttttcggtaatgATTTCACATTTACAGACCTCAACCTACCTCCAACTCTCAC
Klf4 B4 14	ggcactggaaagggcggtgATTTCACATTTACAGACCTCAACCTACCTCCAACTCTCAC

Klf4 B4 15	aaaatgctctgtcacaccgATTTCACATTTACAGACCTCAACCTACCTCCAACTCTCAC
Klf4 B4 16	aaggcgaggtgatccgaccATTTCACATTTACAGACCTCAACCTACCTCCAACTCTCAC
Klf4 B4 17	taaaagtgcctcttcatgtATTTCACATTTACAGACCTCAACCTACCTCCAACTCTCAC
Tfap2A B5 1	ctcgtacttgatgttatccgATTTCACTTCATATCACTCACTCCCAATCTCTATCTACCC
Tfap2A B5 2	ctgagactgggggtagatggATTTCACTTCATATCACTCACTCCCAATCTCTATCTACCC
Tfap2A B5 3	tcgttgacgtgggagtagggATTTCACTTCATATCACTCACTCCCAATCTCTATCTACCC
Tfap2A B5 4	cagggagttgaggctgtaggATTTCACTTCATATCACTCACTCCCAATCTCTATCTACCC
Tfap2A B5 5	atggcgtgagggatggagtgATTTCACTTCATATCACTCACTCCCAATCTCTATCTACCC
Tfap2A B5 6	tctggtccgggatgttgatgATTTCACTTCATATCACTCACTCCCAATCTCTATCTACCC
Tfap2A B5 7	acggggcctttcttaatgacATTTCACTTCATATCACTCACTCCCAATCTCTATCTACCC
Tfap2A B5 8	gttgttagacttggagagggATTTCACTTCATATCACTCACTCCCAATCTCTATCTACCC
Tfap2A B5 9	ttgatggggatggaggagacATTTCACTTCATATCACTCACTCCCAATCTCTATCTACCC
Tfap2A B5 10	catttttggactttgccctcATTTCACTTCATATCACTCACTCCCAATCTCTATCTACCC
Tfap2A B5 11	aggttcaaccctatcttgtcATTTCACTTCATATCACTCACTCCCAATCTCTATCTACCC
Tfap2A B5 12	gtgagcaaggtgacattagcATTTCACTTCATATCACTCACTCCCAATCTCTATCTACCC
Tfap2A B5 13	atctctagctagatgcactgATTTCACTTCATATCACTCACTCCCAATCTCTATCTACCC
Tfap2A B5 14	ttctgtctcacaaacgtaccATTTCACTTCATATCACTCACTCCCAATCTCTATCTACCC
Tfap2A B5 15	aattcagctactgctttggcATTTCACTTCATATCACTCACTCCCAATCTCTATCTACCC
Tfap2A B5 16	atcggaatgttgtcggttgaATTTCACTTCATATCACTCACTCCCAATCTCTATCTACCC
Tfap2A B5 17	tttttcttgtgacttgctcgATTTCACTTCATATCACTCACTCCCAATCTCTATCTACCC
Tfap2A B5 18	ctgttttgtagctagaagcaATTTCACTTCATATCACTCACTCCCAATCTCTATCTACCC
Tfap2A B5 19	ggtcggtgaactctttacagATTTCACTTCATATCACTCACTCCCAATCTCTATCTACCC
Tfap2A B5 20	cggctccaaaaatggggttggATTTCACTTCATATCACTCACTCCCAATCTCTATCTACCC
Tfap2A B5 21	gagatgaggttgaagtgggtATTTCACTTCATATCACTCACTCCCAATCTCTATCTACCC
Tfap2A B5 22	tcggtgagatagttctgcagATTTCACTTCATATCACTCACTCCCAATCTCTATCTACCC
Tfap2A B5 23	gttgctgaggtacattttgtATTTCACTTCATATCACTCACTCCCAATCTCTATCTACCC
Tfap2A B5 24	ctctttgtcgctgcttttggATTTCACTTCATATCACTCACTCCCAATCTCTATCTACCC

Hyb 3

Sox10 B1 1	cgacagatcttggtcatcagTATAGCATTCTTTCTTGAGGAGGGCAGCAAACGGGAAGAG
Sox10 B1 2	gcagtggtggtcttcagaacTATAGCATTCTTTCTTGAGGAGGGCAGCAAACGGGAAGAG
Sox10 B1 3	gtgcgaagagttgtccgatgTATAGCATTCTTTCTTGAGGAGGGCAGCAAACGGGAAGAG
Sox10 B1 4	tgctcttgttacttccattgTATAGCATTCTTTCTTGAGGAGGGCAGCAAACGGGAAGAG
Sox10 B1 5	caaggtcttactgagctcagTATAGCATTCTTTCTTGAGGAGGGCAGCAAACGGGAAGAG
Sox10 B1 6	tgtagtgagcctggatagagTATAGCATTCTTTCTTGAGGAGGGCAGCAAACGGGAAGAG
Sox10 B1 7	tggctctgacctgaagagtgTATAGCATTCTTTCTTGAGGAGGGCAGCAAACGGGAAGAG
Sox10 B1 8	ctcgcttggagtcagctttgTATAGCATTCTTTCTTGAGGAGGGCAGCAAACGGGAAGAG
Sox10 B1 9	tccacattgccaaagtcaatTATAGCATTCTTTCTTGAGGAGGGCAGCAAACGGGAAGAG
Sox10 B1 10	acatcacttcatggctgatcTATAGCATTCTTTCTTGAGGAGGGCAGCAAACGGGAAGAG
Sox10 B1 11	tgacgtcaaaggtctccatgTATAGCATTCTTTCTTGAGGAGGGCAGCAAACGGGAAGAG
Sox10 B1 12	gtggcaggtattggtcaaatTATAGCATTCTTTCTTGAGGAGGGCAGCAAACGGGAAGAG
Sox10 B1 13	ttggagatccaggctgagtgTATAGCATTCTTTCTTGAGGAGGGCAGCAAACGGGAAGAG
Sox10 B1 14	cggacaaggagactccatgcTATAGCATTCTTTCTTGAGGAGGGCAGCAAACGGGAAGAG
Sox10 B1 15	ttttcacctgggctttggagTATAGCATTCTTTCTTGAGGAGGGCAGCAAACGGGAAGAG
Sox10 B1 16	ctgatcggtgtaatgacctcTATAGCATTCTTTCTTGAGGAGGGCAGCAAACGGGAAGAG
Sox10 B1 17	gatgtgtaagctatctgggaTATAGCATTCTTTCTTGAGGAGGGCAGCAAACGGGAAGAG
Sox10 B1 18	aaccatagtggggcagactcTATAGCATTCTTTCTTGAGGAGGGCAGCAAACGGGAAGAG
Sox10 B1 19	ggtggtctggatagtcaaacTATAGCATTCTTTCTTGAGGAGGGCAGCAAACGGGAAGAG
Sox10 B1 20	aaggcagagtagaggccagaTATAGCATTCTTTCTTGAGGAGGGCAGCAAACGGGAAGAG
Sox10 B1 21	ctgggaaggtcccatataggTATAGCATTCTTTCTTGAGGAGGGCAGCAAACGGGAAGAG
Sox10 B1 22	ggtcagagatggcagtgtaaTATAGCATTCTTTCTTGAGGAGGGCAGCAAACGGGAAGAG
Sox10 B1 23	caatgtgtgggactatgggaTATAGCATTCTTTCTTGAGGAGGGCAGCAAACGGGAAGAG
Sox10 B1 24	agagagtcgtatacacaggcTATAGCATTCTTTCTTGAGGAGGGCAGCAAACGGGAAGAG
Pax7 B2 1	gtggacacttccaaagggaaAAAAAGCTCAGTCCATCCTCGTAAATCCTCATCAATCATC
Pax7 B2 2	ttgatgaaaacccctccgagAAAAAGCTCAGTCCATCCTCGTAAATCCTCATCAATCATC
Pax7 B2 3	acgatcttatggcggatgtgAAAAAGCTCAGTCCATCCTCGTAAATCCTCATCAATCATC
Pax7 B2 4	atgcgtagcacacggctaatAAAAAGCTCAGTCCATCCTCGTAAATCCTCATCAATCATC
Pax7 B2 5	aggatgccatctatgctgtgAAAAAGCTCAGTCCATCCTCGTAAATCCTCATCAATCATC
Pax7 B2 6	gttctgattcgacatcggagAAAAAGCTCAGTCCATCCTCGTAAATCCTCATCAATCATC

Pax7 B2 7	tgcttcctcttcaaaggcagAAAAAGCTCAGTCCATCCTCGTAAATCCTCATCAATCATC
Pax7 B2 8	ctgctcggcagtgaaagtggAAAAAGCTCAGTCCATCCTCGTAAATCCTCATCAATCATC
Pax7 B2 9	ctgaaccacacctgaacacgAAAAAGCTCAGTCCATCCTCGTAAATCCTCATCAATCATC
Pax7 B2 10	agatggttgaatgctgcgagAAAAAGCTCAGTCCATCCTCGTAAATCCTCATCAATCATC
Pax7 B2 11	catggtggatggtggcaaggAAAAAGCTCAGTCCATCCTCGTAAATCCTCATCAATCATC
Pax7 B2 12	cataggcagagctggagtcgAAAAAGCTCAGTCCATCCTCGTAAATCCTCATCAATCATC
Pax7 B2 13	agcattcatgaagctgtctgAAAAAGCTCAGTCCATCCTCGTAAATCCTCATCAATCATC
Pax7 B2 14	attcatgtggttggcaggagAAAAAGCTCAGTCCATCCTCGTAAATCCTCATCAATCATC
Pax7 B2 15	gagagaggccattgctaacaAAAAAGCTCAGTCCATCCTCGTAAATCCTCATCAATCATC
Pax7 B2 16	gagcactgcatcttgttttgAAAAAGCTCAGTCCATCCTCGTAAATCCTCATCAATCATC
Pax7 B2 17	tcaaggctatggtgaggttcAAAAAGCTCAGTCCATCCTCGTAAATCCTCATCAATCATC
Pax7 B2 18	aggatgctcatcacctgattAAAAAGCTCAGTCCATCCTCGTAAATCCTCATCAATCATC
Pax7 B2 19	cagagatggagttggtggtgAAAAAGCTCAGTCCATCCTCGTAAATCCTCATCAATCATC
Pax7 B2 20	cacggacttgatggagtcacAAAAAGCTCAGTCCATCCTCGTAAATCCTCATCAATCATC
Pax7 B2 21	tgtaactggtggtgctgtagAAAAAGCTCAGTCCATCCTCGTAAATCCTCATCAATCATC
Pax7 B2 22	gctcacgttcttggtcaaatAAAAAGCTCAGTCCATCCTCGTAAATCCTCATCAATCATC
Pax7 B2 23	cagaacggccgaatgttctcAAAAAGCTCAGTCCATCCTCGTAAATCCTCATCAATCATC
Pax7 B2 24	ctgtctctactggtaggagcAAAAAGCTCAGTCCATCCTCGTAAATCCTCATCAATCATC
Nanog B3 1	ctaacagagccgtaggacggTAAAAAAGTCTAATCCGTCCCTGCCTCTATATCTCCACTC
Nanog B3 2	ttttctgctctgtggtcaagTAAAAAAGTCTAATCCGTCCCTGCCTCTATATCTCCACTC
Nanog B3 3	gaagaagctggagaggcatcTAAAAAAGTCTAATCCGTCCCTGCCTCTATATCTCCACTC
Nanog B3 4	gtatactgaatgagtgtcccTAAAAAAGTCTAATCCGTCCCTGCCTCTATATCTCCACTC
Nanog B3 5	cagtagggctagtggcagagTAAAAAAGTCTAATCCGTCCCTGCCTCTATATCTCCACTC
Nanog B3 6	aaaagtggggcggtgagatgTAAAAAAGTCTAATCCGTCCCTGCCTCTATATCTCCACTC
Nanog B3 7	ctctcacctttatccttaacTAAAAAAGTCTAATCCGTCCCTGCCTCTATATCTCCACTC
Nanog B3 8	agaaagctgtgcggctcttgTAAAAAAGTCTAATCCGTCCCTGCCTCTATATCTCCACTC
Nanog B3 9	gataaatccctttatccaccTAAAAAAGTCTAATCCGTCCCTGCCTCTATATCTCCACTC
Nanog B3 10	tgatgaaacccattctgtggTAAAAAAGTCTAATCCGTCCCTGCCTCTATATCTCCACTC
Nanog B3 11	ggtcatatccagatacgcagTAAAAAAGTCTAATCCGTCCCTGCCTCTATATCTCCACTC
Nanog B3 12	ggaagccctggtgaaatgtaTAAAAAAGTCTAATCCGTCCCTGCCTCTATATCTCCACTC
Nanog B3 13	gaaggtttctgttggcaacaTAAAAAAGTCTAATCCGTCCCTGCCTCTATATCTCCACTC
Nanog B3 14	aagtctggccactgctgtaaTAAAAAAGTCTAATCCGTCCCTGCCTCTATATCTCCACTC
Nanog B3 15	cacagccatgaacggatacaTAAAAAAGTCTAATCCGTCCCTGCCTCTATATCTCCACTC
Nanog B3 16	gttccaccttttccaaagaaTAAAAAAGTCTAATCCGTCCCTGCCTCTATATCTCCACTC
Nanog B3 17	gacttaataaacccatggccTAAAAAAGTCTAATCCGTCCCTGCCTCTATATCTCCACTC
Nanog B3 18	gccatgatagaagttcatctTAAAAAAGTCTAATCCGTCCCTGCCTCTATATCTCCACTC
Nanog B3 19	tgtcataatccacattggtaTAAAAAAGTCTAATCCGTCCCTGCCTCTATATCTCCACTC
Nanog B3 20	taggtatcttctgcctgcaaTAAAAAAGTCTAATCCGTCCCTGCCTCTATATCTCCACTC
Nanog B3 21	tatcagaggtgctctggaagTAAAAAAGTCTAATCCGTCCCTGCCTCTATATCTCCACTC
Nanog B3 22	gagctcgagaactgtgtgatTAAAAAAGTCTAATCCGTCCCTGCCTCTATATCTCCACTC
Nanog B3 23	ctggtactgatgccgtacagTAAAAAAGTCTAATCCGTCCCTGCCTCTATATCTCCACTC
Nanog B3 24	agtctcataaccattctgggTAAAAAAGTCTAATCCGTCCCTGCCTCTATATCTCCACTC
Sox2 B4 1	ttcagctcggtttccatcatATTTCACATTTACAGACCTCAACCTACCTCCAACTCTCAC
Sox2 B4 2	tgttcttctggttgttcgcgATTTCACATTTACAGACCTCAACCTACCTCCAACTCTCAC
Sox2 B4 3	ataaaagtttccactcggccATTTCACATTTACAGACCTCAACCTACCTCCAACTCTCAC
Sox2 B4 4	cttcgtcaatgaagggtctcATTTCACATTTACAGACCTCAACCTACCTCCAACTCTCAC
Sox2 B4 5	ttcatgtgcagagctcggagATTTCACATTTACAGACCTCAACCTACCTCCAACTCTCAC
Sox2 B4 6	ttcatcagggtcttggttttATTTCACATTTACAGACCTCAACCTACCTCCAACTCTCAC
Sox2 B4 7	ctggcaacgtgtacttatccATTTCACATTTACAGACCTCAACCTACCTCCAACTCTCAC
Sox2 B4 8	cggtcgtcatggtattggtgATTTCACATTTACAGACCTCAACCTACCTCCAACTCTCAC
Sox2 B4 9	ttcatgtgcgcgtaactgtcATTTCACATTTACAGACCTCAACCTACCTCCAACTCTCAC
Sox2 B4 10	ctggtcatggagttgtactgATTTCACATTTACAGACCTCAACCTACCTCCAACTCTCAC
Sox2 B4 11	gatccgttcatgtacgtctgATTTCACATTTACAGACCTCAACCTACCTCCAACTCTCAC
Sox2 B4 12	gtacgacatgctgtaggtagATTTCACATTTACAGACCTCAACCTACCTCCAACTCTCAC
Sox2 B4 13	gagctggattccgtcttgacATTTCACATTTACAGACCTCAACCTACCTCCAACTCTCAC
Sox2 B4 14	ctcgaatgagacgaggaggtATTTCACATTTACAGACCTCAACCTACCTCCAACTCTCAC
Sox2 B4 15	gatacatgctgatcatgtccATTTCACATTTACAGACCTCAACCTACCTCCAACTCTCAC
Sox2 B4 16	tggttctggtacttcagcacATTTCACATTTACAGACCTCAACCTACCTCCAACTCTCAC
Sox2 B4 17	catatgaagtctgcttggggATTTCACATTTACAGACCTCAACCTACCTCCAACTCTCAC

Sox2 B4 18	gtgcactctggtagtgttggATTTCACATTTACAGACCTCAACCTACCTCCAACTCTCAC
Sox2 B4 19	ttaatggctgtgccaggaacATTTCACATTTACAGACCTCAACCTACCTCCAACTCTCAC
Sox2 B4 20	atgtgatagagggagtgtgcATTTCACATTTACAGACCTCAACCTACCTCCAACTCTCAC
Snai2 B5 1	gtttcttgaccaggaaggagATTTCACTTCATATCACTCACTCCCAATCTCTATCTACCC
Snai2 B5 2	tagtttggcttcttggatgaATTTCACTTCATATCACTCACTCCCAATCTCTATCTACCC
Snai2 B5 3	atcactgtatgagtgtccagATTTCACTTCATATCACTCACTCCCAATCTCTATCTACCC
Snai2 B5 4	agctttcatacaggtatgggATTTCACTTCATATCACTCACTCCCAATCTCTATCTACCC
Snai2 B5 5	ctgcggtatgatagggactgATTTCACTTCATATCACTCACTCCCAATCTCTATCTACCC
Snai2 B5 6	tgtaagctacagagctcaggATTTCACTTCATATCACTCACTCCCAATCTCTATCTACCC
Snai2 B5 7	gtagtccacacagtaatgggATTTCACTTCATATCACTCACTCCCAATCTCTATCTACCC
Snai2 B5 8	attgggtagaggagacggtaATTTCACTTCATATCACTCACTCCCAATCTCTATCTACCC
Snai2 B5 9	atccagaaagcggagagaggATTTCACTTCATATCACTCACTCCCAATCTCTATCTACCC
Snai2 B5 10	tgacccttcccaaagatgagATTTCACTTCATATCACTCACTCCCAATCTCTATCTACCC
Snai2 B5 11	tgatctttggaggaggtgtcATTTCACTTCATATCACTCACTCCCAATCTCTATCTACCC
Snai2 B5 12	aatgggactttctgaaccgcATTTCACTTCATATCACTCACTCCCAATCTCTATCTACCC
Snai2 B5 13	atgagggtctgaaagcttggATTTCACTTCATATCACTCACTCCCAATCTCTATCTACCC
Snai2 B5 14	ctgaaacttttcggcttcgaATTTCACTTCATATCACTCACTCCCAATCTCTATCTACCC
Snai2 B5 15	tttggccaacccagagaaagATTTCACTTCATATCACTCACTCCCAATCTCTATCTACCC
Snai2 B5 16	aggctgacatactccttgtcATTTCACTTCATATCACTCACTCCCAATCTCTATCTACCC
Snai2 B5 17	atcttgcagacgcaaggtagATTTCACTTCATATCACTCACTCCCAATCTCTATCTACCC
Snai2 B5 18	agggtctggagaaagccttgATTTCACTTCATATCACTCACTCCCAATCTCTATCTACCC
Snai2 B5 19	ctgatgtgcccttgaagtagATTTCACTTCATATCACTCACTCCCAATCTCTATCTACCC
Snai2 B5 20	gaaagccctgttgcagtgagATTTCACTTCATATCACTCACTCCCAATCTCTATCTACCC
Snai2 B5 21	atgagccctcagattggatcATTTCACTTCATATCACTCACTCCCAATCTCTATCTACCC
Snai2 B5 22	gtatttcttcacatccgagtATTTCACTTCATATCACTCACTCCCAATCTCTATCTACCC
Snai2 B5 23	gcagaagagacattctggagATTTCACTTCATATCACTCACTCCCAATCTCTATCTACCC
Snai2 B5 24	cagccagattcctcatgtttATTTCACTTCATATCACTCACTCCCAATCTCTATCTACCC

Hyb 4

MycN B1 1	cgggttcttgctgatcattcTATAGCATTCTTTCTTGAGGAGGGCAGCAAACGGGAAGAG
MycN B1 2	ggctgtaaggaatcgaactcTATAGCATTCTTTCTTGAGGAGGGCAGCAAACGGGAAGAG
MycN B1 3	atcttcgtccgggtagaaacTATAGCATTCTTTCTTGAGGAGGGCAGCAAACGGGAAGAG
MycN B1 4	ccggcccgcataaatagaaaTATAGCATTCTTTCTTGAGGAGGGCAGCAAACGGGAAGAG
MycN B1 5	caggaggagcaactcggacgTATAGCATTCTTTCTTGAGGAGGGCAGCAAACGGGAAGAG
MycN B1 6	aggcccgtctcgaagaaatcTATAGCATTCTTTCTTGAGGAGGGCAGCAAACGGGAAGAG
MycN B1 7	ggagttgagattgttgctcgTATAGCATTCTTTCTTGAGGAGGGCAGCAAACGGGAAGAG
MycN B1 8	ttgacggggaaggggaagacTATAGCATTCTTTCTTGAGGAGGGCAGCAAACGGGAAGAG
MycN B1 9	ctccactgtcacaacatctaTATAGCATTCTTTCTTGAGGAGGGCAGCAAACGGGAAGAG
MycN B1 10	tgttggaggaggagcgtcttTATAGCATTCTTTCTTGAGGAGGGCAGCAAACGGGAAGAG
MycN B1 11	atagtgagggtggtaacagcTATAGCATTCTTTCTTGAGGAGGGCAGCAAACGGGAAGAG
MycN B1 12	ggtatttttaggacgcactgTATAGCATTCTTTCTTGAGGAGGGCAGCAAACGGGAAGAG
MycN B1 13	gtgtcctgaccgatggaaaaTATAGCATTCTTTCTTGAGGAGGGCAGCAAACGGGAAGAG
MycN B1 14	ataattatgctgctggtgaaTATAGCATTCTTTCTTGAGGAGGGCAGCAAACGGGAAGAG
MycN B1 15	ccatatacggagaaggagcgTATAGCATTCTTTCTTGAGGAGGGCAGCAAACGGGAAGAG
MycN B1 16	cacctcggcttttaacttttTATAGCATTCTTTCTTGAGGAGGGCAGCAAACGGGAAGAG
MycN B1 17	tgaactcttagactttggttTATAGCATTCTTTCTTGAGGAGGGCAGCAAACGGGAAGAG
MycN B1 18	ttatggttacgtcgacgttcTATAGCATTCTTTCTTGAGGAGGGCAGCAAACGGGAAGAG
MycN B1 19	agatcattacgcctctgacgTATAGCATTCTTTCTTGAGGAGGGCAGCAAACGGGAAGAG
MycN B1 20	taacgtgaggaaactggaccTATAGCATTCTTTCTTGAGGAGGGCAGCAAACGGGAAGAG
MycN B1 21	acaagttctggaacgtggtcTATAGCATTCTTTCTTGAGGAGGGCAGCAAACGGGAAGAG
MycN B1 22	cacaacttttgcagctttctTATAGCATTCTTTCTTGAGGAGGGCAGCAAACGGGAAGAG
MycN B1 23	ggacatattcagtggcttttTATAGCATTCTTTCTTGAGGAGGGCAGCAAACGGGAAGAG
MycN B1 24	aagtccgcttgtactctattTATAGCATTCTTTCTTGAGGAGGGCAGCAAACGGGAAGAG
Ets1 B2 1	tcttgatgatggtcagggtgAAAAAGCTCAGTCCATCCTCGTAAATCCTCATCAATCATC
Ets1 B2 2	ggacgggaagagatcgatgtAAAAAGCTCAGTCCATCCTCGTAAATCCTCATCAATCATC
Ets1 B2 3	agtgcctgagacatcatttcAAAAAGCTCAGTCCATCCTCGTAAATCCTCATCAATCATC
Ets1 B2 4	ctttgcgaagccactgaaggAAAAAGCTCAGTCCATCCTCGTAAATCCTCATCAATCATC
Ets1 B2 5	ctggggatctttggggattcAAAAAGCTCAGTCCATCCTCGTAAATCCTCATCAATCATC
Ets1 B2 6	tgaactcattcactgcccacAAAAAGCTCAGTCCATCCTCGTAAATCCTCATCAATCATC

Ets1 B2 7	cagctccgttcatgcagaacAAAAAGCTCAGTCCATCCTCGTAAATCCTCATCAATCATC
Ets1 B2 8	acaaagtcaggccgtagctcAAAAAGCTCAGTCCATCCTCGTAAATCCTCATCAATCATC
Ets1 B2 9	aggtgttcccaaaggatatcAAAAAGCTCAGTCCATCCTCGTAAATCCTCATCAATCATC
Ets1 B2 10	tctggatacgctgcattcacAAAAAGCTCAGTCCATCCTCGTAAATCCTCATCAATCATC
Ets1 B2 11	gtagtctgaagtatagcgggAAAAAGCTCAGTCCATCCTCGTAAATCCTCATCAATCATC
Ets1 B2 12	gtgctcgatgccataactaaAAAAAGCTCAGTCCATCCTCGTAAATCCTCATCAATCATC
Ets1 B2 13	ctgagggatagtcgttctcgAAAAAGCTCAGTCCATCCTCGTAAATCCTCATCAATCATC
Ets1 B2 14	agtagtctgtctgcagggagAAAAAGCTCAGTCCATCCTCGTAAATCCTCATCAATCATC
Ets1 B2 15	cagtttacctcgactggcacAAAAAGCTCAGTCCATCCTCGTAAATCCTCATCAATCATC
Ets1 B2 16	aggctctggaaggaggactgAAAAAGCTCAGTCCATCCTCGTAAATCCTCATCAATCATC
Ets1 B2 17	tcctctgagtcaaagctatcAAAAAGCTCAGTCCATCCTCGTAAATCCTCATCAATCATC
Ets1 B2 18	cgatctcgaacatagtccttAAAAAGCTCAGTCCATCCTCGTAAATCCTCATCAATCATC
Ets1 B2 19	gaatgacaggcttgtccttgAAAAAGCTCAGTCCATCCTCGTAAATCCTCATCAATCATC
Ets1 B2 20	cgtccagcttatgaaggactAAAAAGCTCAGTCCATCCTCGTAAATCCTCATCAATCATC
Ets1 B2 21	acctcatctggatcggaaagAAAAAGCTCAGTCCATCCTCGTAAATCCTCATCAATCATC
Ets1 B2 22	tgggcttgtttttcctcttgAAAAAGCTCAGTCCATCCTCGTAAATCCTCATCAATCATC
Ets1 B2 23	cgtaatagtaacgcagaccaAAAAAGCTCAGTCCATCCTCGTAAATCCTCATCAATCATC
Ets1 B2 24	cagacgaagcggtagacgtaAAAAAGCTCAGTCCATCCTCGTAAATCCTCATCAATCATC
Pax6 B3 1	cgagttccacgatcttctgcTAAAAAAGTCTAATCCGTCCCTGCCTCTATATCTCCACTC
Pax6 B3 2	tccattcgacacgttttgatTAAAAAAGTCTAATCCGTCCCTGCCTCTATATCTCCACTC
Pax6 B3 3	cctgcccaaaattttactcaTAAAAAAGTCTAATCCGTCCCTGCCTCTATATCTCCACTC
Pax6 B3 4	tgatggagccagtttcgtaaTAAAAAAGTCTAATCCGTCCCTGCCTCTATATCTCCACTC
Pax6 B3 5	tactctcggcttactacctcTAAAAAAGTCTAATCCGTCCCTGCCTCTATATCTCCACTC
Pax6 B3 6	tctcgtttatactgcgctatTAAAAAAGTCTAATCCGTCCCTGCCTCTATATCTCCACTC
Pax6 B3 7	tctcccacgcaaagatggagTAAAAAAGTCTAATCCGTCCCTGCCTCTATATCTCCACTC
Pax6 B3 8	tccgagagtaatctgtctcgTAAAAAAGTCTAATCCGTCCCTGCCTCTATATCTCCACTC
Pax6 B3 9	ggactctgtttatcgacgacTAAAAAAGTCTAATCCGTCCCTGCCTCTATATCTCCACTC
Pax6 B3 10	catctgttgcttttcgctagTAAAAAAGTCTAATCCGTCCCTGCCTCTATATCTCCACTC
Pax6 B3 11	atccttagcttgtcgtacatTAAAAAAGTCTAATCCGTCCCTGCCTCTATATCTCCACTC
Pax6 B3 12	catcggaatcttccccattgTAAAAAAGTCTAATCCGTCCCTGCCTCTATATCTCCACTC
Pax6 B3 13	ttcgatttgctcttgggtaaTAAAAAAGTCTAATCCGTCCCTGCCTCTATATCTCCACTC
Pax6 B3 14	tctcgcaaacacatcaggatTAAAAAAGTCTAATCCGTCCCTGCCTCTATATCTCCACTC
Pax6 B3 15	ctattttagcagctagtcgcTAAAAAAGTCTAATCCGTCCCTGCCTCTATATCTCCACTC
Pax6 B3 16	ttggcccttctgttagaaaaTAAAAAAGTCTAATCCGTCCCTGCCTCTATATCTCCACTC
Pax6 B3 17	aactactgctgatggggatgTAAAAAAGTCTAATCCGTCCCTGCCTCTATATCTCCACTC
Pax6 B3 18	tgtgggatcggctggtaaacTAAAAAAGTCTAATCCGTCCCTGCCTCTATATCTCCACTC
Pax6 B3 19	aagaggaaacgggggtggtcTAAAAAAGTCTAATCCGTCCCTGCCTCTATATCTCCACTC
Pax6 B3 20	cccaacatggaacctgatgtTAAAAAAGTCTAATCCGTCCCTGCCTCTATATCTCCACTC
Pax6 B3 21	gttgcataggcaggttgttgTAAAAAAGTCTAATCCGTCCCTGCCTCTATATCTCCACTC
Pax6 B3 22	ctattcatgtgtgtctgcatTAAAAAAGTCTAATCCGTCCCTGCCTCTATATCTCCACTC
Pax6 B3 23	agacatgtcaggttcactgcTAAAAAAGTCTAATCCGTCCCTGCCTCTATATCTCCACTC
Pax6 B3 24	actgtaatcttggccaatacTAAAAAAGTCTAATCCGTCCCTGCCTCTATATCTCCACTC
PouV B4 1	ccatttacacattcgcagcaATTTCACATTTACAGACCTCAACCTACCTCCAACTCTCAC
PouV B4 2	gggcatttcgcaatcctttaATTTCACATTTACAGACCTCAACCTACCTCCAACTCTCAC
PouV B4 3	gagataccgcgggtgacaacATTTCACATTTACAGACCTCAACCTACCTCCAACTCTCAC
PouV B4 4	aggcagacacgcggtgaatgATTTCACATTTACAGACCTCAACCTACCTCCAACTCTCAC
PouV B4 5	ttgaggtccttggcaaactgATTTCACATTTACAGACCTCAACCTACCTCCAACTCTCAC
PouV B4 6	gagtgaagcccagcatgatgATTTCACATTTACAGACCTCAACCTACCTCCAACTCTCAC
PouV B4 7	tgaacatcttcccatagagcATTTCACATTTACAGACCTCAACCTACCTCCAACTCTCAC
PouV B4 8	tgagctggagagcttcgaagATTTCACATTTACAGACCTCAACCTACCTCCAACTCTCAC
PouV B4 9	tcattgagccaacgctgcagATTTCACATTTACAGACCTCAACCTACCTCCAACTCTCAC
PouV B4 10	catgttgtccgtgttctctgATTTCACATTTACAGACCTCAACCTACCTCCAACTCTCAC
PouV B4 11	acttgctctgcattgcacatATTTCACATTTACAGACCTCAACCTACCTCCAACTCTCAC
PouV B4 12	tcacgttggtctcgatgctgATTTCACATTTACAGACCTCAACCTACCTCCAACTCTCAC
PouV B4 13	gaagaagctctccagcgttcATTTCACATTTACAGACCTCAACCTACCTCCAACTCTCAC
PouV B4 14	gactgggcttcacacatttgATTTCACATTTACAGACCTCAACCTACCTCCAACTCTCAC
PouV B4 15	tcagcgatctgggagatctcATTTCACATTTACAGACCTCAACCTACCTCCAACTCTCAC
PouV B4 16	aacgtctttgtccaggttgaATTTCACATTTACAGACCTCAACCTACCTCCAACTCTCAC
PouV B4 17	tggttcatgtcgtacatcacATTTCACATTTACAGACCTCAACCTACCTCCAACTCTCAC

PouV B4 18	ctgggatgtcactgggatggATTTCACATTTACAGACCTCAACCTACCTCCAACTCTCAC
PouV B4 19	gaaaggtggcatgtagacggATTTCACATTTACAGACCTCAACCTACCTCCAACTCTCAC
PouV B4 20	tgctgttgttcatggagatcATTTCACATTTACAGACCTCAACCTACCTCCAACTCTCAC
Msx1 B5 1	cggtggtcatgtccgcagATTTCACTTCATATCACTCACTCCCAATCTCTATCTACCC
Msx1 B5 2	ttgctgaaggcggaggcgATTTCACTTCATATCACTCACTCCCAATCTCTATCTACCC
Msx1 B5 3	tgggtttgtcgctctcctATTTCACTTCATATCACTCACTCCCAATCTCTATCTACCC
Msx1 B5 4	cagcggggaaggggacacATTTCACTTCATATCACTCACTCCCAATCTCTATCTACCC
Msx1 B5 5	agcgcttccacgctgaagATTTCACTTCATATCACTCACTCCCAATCTCTATCTACCC
Msx1 B5 6	gtcagagcgccgaggttgATTTCACTTCATATCACTCACTCCCAATCTCTATCTACCC
Msx1 B5 7	cgagaggcagcggcgacgATTTCACTTCATATCACTCACTCCCAATCTCTATCTACCC
Msx1 B5 8	cgacggacgggaagtggcATTTCACTTCATATCACTCACTCCCAATCTCTATCTACCC
Msx1 B5 9	tctctgctttgagcagcgATTTCACTTCATATCACTCACTCCCAATCTCTATCTACCC
Msx1 B5 10	cgagaagcgggggctctgATTTCACTTCATATCACTCACTCCCAATCTCTATCTACCC
Msx1 B5 11	tgttggtcttgtgcttgcATTTCACTTCATATCACTCACTCCCAATCTCTATCTACCC
Msx1 B5 12	agctggagaactcggcacATTTCACTTCATATCACTCACTCCCAATCTCTATCTACCC
Msx1 B5 13	gaatgcagcaggcgggagATTTCACTTCATATCACTCACTCCCAATCTCTATCTACCC
Msx1 B5 14	gcccaacgggaaggagatATTTCACTTCATATCACTCACTCCCAATCTCTATCTACCC
Msx1 B5 15	ctccgtacagggatgcgcATTTCACTTCATATCACTCACTCCCAATCTCTATCTACCC
Msx1 B5 16	tcgctggaaggggctggaATTTCACTTCATATCACTCACTCCCAATCTCTATCTACCC
Msx1 B5 17	gtgtacagtcccacagggATTTCACTTCATATCACTCACTCCCAATCTCTATCTACCC
Msx1 B5 18	tactatatcccacgtgcgATTTCACTTCATATCACTCACTCCCAATCTCTATCTACCC

Hyb 5

Axud1 B1 1	gagcagtaggtgctgtcatcTATAGCATTCTTTCTTGAGGAGGGCAGCAAACGGGAAGAG
Axud1 B1 2	ggtaaagccaggtgctatggTATAGCATTCTTTCTTGAGGAGGGCAGCAAACGGGAAGAG
Axud1 B1 3	cgtttggatttcttcaggatTATAGCATTCTTTCTTGAGGAGGGCAGCAAACGGGAAGAG
Axud1 B1 4	caaactccacgttgttcttcTATAGCATTCTTTCTTGAGGAGGGCAGCAAACGGGAAGAG
Axud1 B1 5	gcgtgggaagtagaacacagTATAGCATTCTTTCTTGAGGAGGGCAGCAAACGGGAAGAG
Axud1 B1 6	gaactctgctaacgtgaactTATAGCATTCTTTCTTGAGGAGGGCAGCAAACGGGAAGAG
Axud1 B1 7	cgacgaatgttttcctgctcTATAGCATTCTTTCTTGAGGAGGGCAGCAAACGGGAAGAG
Axud1 B1 8	tttgtgccattcatggtgagTATAGCATTCTTTCTTGAGGAGGGCAGCAAACGGGAAGAG
Axud1 B1 9	gagatgtcctcaatggtgagTATAGCATTCTTTCTTGAGGAGGGCAGCAAACGGGAAGAG
Axud1 B1 10	cattgctgacgtcaatgtcaTATAGCATTCTTTCTTGAGGAGGGCAGCAAACGGGAAGAG
Axud1 B1 11	tggaggaaaaagccatcctcTATAGCATTCTTTCTTGAGGAGGGCAGCAAACGGGAAGAG
Axud1 B1 12	cctccttgtcaatcttcttcTATAGCATTCTTTCTTGAGGAGGGCAGCAAACGGGAAGAG
Axud1 B1 13	agacctcctgacagtcacagTATAGCATTCTTTCTTGAGGAGGGCAGCAAACGGGAAGAG
Axud1 B1 14	ttgtacagccacaggggaagTATAGCATTCTTTCTTGAGGAGGGCAGCAAACGGGAAGAG
Axud1 B1 15	ctggttgaactcgatcctgcTATAGCATTCTTTCTTGAGGAGGGCAGCAAACGGGAAGAG
Axud1 B1 16	aagccatcgtcatcgatgtcTATAGCATTCTTTCTTGAGGAGGGCAGCAAACGGGAAGAG
Axud1 B1 17	atcgttgaagtggaggatgcTATAGCATTCTTTCTTGAGGAGGGCAGCAAACGGGAAGAG
Axud1 B1 18	actcagagaggtgggatgagTATAGCATTCTTTCTTGAGGAGGGCAGCAAACGGGAAGAG
Axud1 B1 19	tagctcttggaacacgactcTATAGCATTCTTTCTTGAGGAGGGCAGCAAACGGGAAGAG
Axud1 B1 20	gtccgagaaggactggaagaTATAGCATTCTTTCTTGAGGAGGGCAGCAAACGGGAAGAG
Axud1 B1 21	agttgtaaaggggtcccaagTATAGCATTCTTTCTTGAGGAGGGCAGCAAACGGGAAGAG
Axud1 B1 22	tcaaggttctcatactccttTATAGCATTCTTTCTTGAGGAGGGCAGCAAACGGGAAGAG
Axud1 B1 23	ctgaaactgtaacgctgagaTATAGCATTCTTTCTTGAGGAGGGCAGCAAACGGGAAGAG
Axud1 B1 24	gcgggaaaccggggaaattaTATAGCATTCTTTCTTGAGGAGGGCAGCAAACGGGAAGAG
Tfap2B B2 1	acattttcaaccagtttccaAAAAAGCTCAGTCCATCCTCGTAAATCCTCATCAATCATC
Tfap2B B2 2	gtcctcatagatatcttcgtAAAAAGCTCAGTCCATCCTCGTAAATCCTCATCAATCATC
Tfap2B B2 3	gaggacggggtgtgagagagAAAAAGCTCAGTCCATCCTCGTAAATCCTCATCAATCATC
Tfap2B B2 4	tagggggggtgggaaataaggAAAAAGCTCAGTCCATCCTCGTAAATCCTCATCAATCATC
Tfap2B B2 5	tggctttggtggtaaggaagAAAAAGCTCAGTCCATCCTCGTAAATCCTCATCAATCATC
Tfap2B B2 6	ggtcattgacatgggagtaaAAAAAGCTCAGTCCATCCTCGTAAATCCTCATCAATCATC
Tfap2B B2 7	gttgatgcaaggggtttaggAAAAAGCTCAGTCCATCCTCGTAAATCCTCATCAATCATC
Tfap2B B2 8	gttcataccgctgttattggAAAAAGCTCAGTCCATCCTCGTAAATCCTCATCAATCATC
Tfap2B B2 9	ttttaatgacagactggtccAAAAAGCTCAGTCCATCCTCGTAAATCCTCATCAATCATC
Tfap2B B2 10	gccatctttattcatcatcaAAAAAGCTCAGTCCATCCTCGTAAATCCTCATCAATCATC
Tfap2B B2 11	ggtgttgacggaaattccacAAAAAGCTCAGTCCATCCTCGTAAATCCTCATCAATCATC
Tfap2B B2 12	ccactgttactttgtacttgAAAAAGCTCAGTCCATCCTCGTAAATCCTCATCAATCATC

Tfap2B B2 13	tagcctttctcgcaaagatcAAAAAGCTCAGTCCATCCTCGTAAATCCTCATCAATCATC
Tfap2B B2 14	aaagtgacatttgcagccttAAAAAGCTCAGTCCATCCTCGTAAATCCTCATCAATCATC
Tfap2B B2 15	tttttcctggagtggaggtcAAAAAGCTCAGTCCATCCTCGTAAATCCTCATCAATCATC
Tfap2B B2 16	aagttgctttgtagcaagcaAAAAAGCTCAGTCCATCCTCGTAAATCCTCATCAATCATC
Tfap2B B2 17	ggccaagagatctgtaaattAAAAAGCTCAGTCCATCCTCGTAAATCCTCATCAATCATC
Tfap2B B2 18	tgtttccaatgggtgtcctgAAAAAGCTCAGTCCATCCTCGTAAATCCTCATCAATCATC
Tfap2B B2 19	ggttccaaaataggggtgggAAAAAGCTCAGTCCATCCTCGTAAATCCTCATCAATCATC
Tfap2B B2 20	cagtcaggtagttttggaggAAAAAGCTCAGTCCATCCTCGTAAATCCTCATCAATCATC
Tfap2B B2 21	atcttgtccatgcctttgagAAAAAGCTCAGTCCATCCTCGTAAATCCTCATCAATCATC
Tfap2B B2 22	tgttagcagtgttgttgttcAAAAAGCTCAGTCCATCCTCGTAAATCCTCATCAATCATC
Tfap2B B2 23	agttttactacctggtccttAAAAAGCTCAGTCCATCCTCGTAAATCCTCATCAATCATC
Tfap2B B2 24	attttctgtgtttctcttccAAAAAGCTCAGTCCATCCTCGTAAATCCTCATCAATCATC
Sox8 B3 1	cctcggtcatgttgagcatTAAAAAAGTCTAATCCGTCCCTGCCTCTATATCTCCACTC
Sox8 B3 2	cacgtgggacatggagctgTAAAAAAGTCTAATCCGTCCCTGCCTCTATATCTCCACTC
Sox8 B3 3	cttcagcacctgcgagacgTAAAAAAGTCTAATCCGTCCCTGCCTCTATATCTCCACTC
Sox8 B3 4	tgtgcaggtgcgggtattgTAAAAAAGTCTAATCCGTCCCTGCCTCTATATCTCCACTC
Sox8 B3 5	agctttcacgcttttcctcTAAAAAAGTCTAATCCGTCCCTGCCTCTATATCTCCACTC
Sox8 B3 6	catgatggctgagctcggcTAAAAAAGTCTAATCCGTCCCTGCCTCTATATCTCCACTC
Sox8 B3 7	tgtcagccttgtagatctgTAAAAAAGTCTAATCCGTCCCTGCCTCTATATCTCCACTC
Sox8 B3 8	aagtcaatgttctggcggcTAAAAAAGTCTAATCCGTCCCTGCCTCTATATCTCCACTC
Sox8 B3 9	ctggtcaaactcgtgcacaTAAAAAAGTCTAATCCGTCCCTGCCTCTATATCTCCACTC
Sox8 B3 10	atggcagtatggccattgaTAAAAAAGTCTAATCCGTCCCTGCCTCTATATCTCCACTC
Sox8 B3 11	gagtaggacgtgctgtagaTAAAAAAGTCTAATCCGTCCCTGCCTCTATATCTCCACTC
Sox8 B3 12	tcttgtgagtccacacctgTAAAAAAGTCTAATCCGTCCCTGCCTCTATATCTCCACTC
Sox8 B3 13	aatcagccgatgacggcgaTAAAAAAGTCTAATCCGTCCCTGCCTCTATATCTCCACTC
Sox8 B3 14	ttttgatgtgcggcctttgTAAAAAAGTCTAATCCGTCCCTGCCTCTATATCTCCACTC
Sox8 B3 15	gggactggtcgctgtagtgTAAAAAAGTCTAATCCGTCCCTGCCTCTATATCTCCACTC
Sox8 B3 16	gtaggagccgtagtcagagTAAAAAAGTCTAATCCGTCCCTGCCTCTATATCTCCACTC
Sox8 B3 17	ggggttgtagtagttggagTAAAAAAGTCTAATCCGTCCCTGCCTCTATATCTCCACTC
Sox8 B3 18	gtagatgctggaggggtaaTAAAAAAGTCTAATCCGTCCCTGCCTCTATATCTCCACTC
Sox8 B3 19	ccgtgaggagtggaagtagTAAAAAAGTCTAATCCGTCCCTGCCTCTATATCTCCACTC
Sox8 B3 20	gacaagccgttgaggatggTAAAAAAGTCTAATCCGTCCCTGCCTCTATATCTCCACTC
Sox8 B3 21	tcgtcagggttgtgtagacTAAAAAAGTCTAATCCGTCCCTGCCTCTATATCTCCACTC
E2f1 B4 1	cgagacgatgaggaggtgcATTTCACATTTACAGACCTCAACCTACCTCCAACTCTCAC
E2f1 B4 2	tggcgaagagcaggaggtcATTTCACATTTACAGACCTCAACCTACCTCCAACTCTCAC
E2f1 B4 3	aagttcagcttccgcttcaATTTCACATTTACAGACCTCAACCTACCTCCAACTCTCAC
E2f1 B4 4	cggctatgtactggtgatcATTTCACATTTACAGACCTCAACCTACCTCCAACTCTCAC
E2f1 B4 5	tttcatagcgggacttctcATTTCACATTTACAGACCTCAACCTACCTCCAACTCTCAC
E2f1 B4 6	gcttggtggtgaggttcagATTTCACATTTACAGACCTCAACCTACCTCCAACTCTCAC
E2f1 B4 7	cagttgaggtccaccacacATTTCACATTTACAGACCTCAACCTACCTCCAACTCTCAC
E2f1 B4 8	aggacattggtgatgtcgtATTTCACATTTACAGACCTCAACCTACCTCCAACTCTCAC
E2f1 B4 9	ttggtgatgagctggatgcATTTCACATTTACAGACCTCAACCTACCTCCAACTCTCAC
E2f1 B4 10	ccactggatgttgttcttgATTTCACATTTACAGACCTCAACCTACCTCCAACTCTCAC
E2f1 B4 11	tggagggatcctcagtgagATTTCACATTTACAGACCTCAACCTACCTCCAACTCTCAC
E2f1 B4 12	gtgccttgataaccatcacATTTCACATTTACAGACCTCAACCTACCTCCAACTCTCAC
E2f1 B4 13	agacttgcagctgggtctcATTTCACATTTACAGACCTCAACCTACCTCCAACTCTCAC
E2f1 B4 14	gggacagaggaacacgtcgATTTCACATTTACAGACCTCAACCTACCTCCAACTCTCAC
E2f1 B4 15	cttgaaggggctcttgacgATTTCACATTTACAGACCTCAACCTACCTCCAACTCTCAC
E2f1 B4 16	gtgtgtccatggaggctagATTTCACATTTACAGACCTCAACCTACCTCCAACTCTCAC
E2f1 B4 17	gaaatcgtccttgccatgcATTTCACATTTACAGACCTCAACCTACCTCCAACTCTCAC
E2f1 B4 18	gaactcatccgccaggaagATTTCACATTTACAGACCTCAACCTACCTCCAACTCTCAC
E2f1 B4 19	aaagagctcactgatgcccATTTCACATTTACAGACCTCAACCTACCTCCAACTCTCAC
Ccnd1 B5 1	tgtcattgagcaggttggcgATTTCACTTCATATCACTCACTCCCAATCTCTATCTACCC
Ccnd1 B5 2	cacttgaagtaggacaccgaATTTCACTTCATATCACTCACTCCCAATCTCTATCTACCC
Ccnd1 B5 3	atccaagtggcgactattttATTTCACTTCATATCACTCACTCCCAATCTCTATCTACCC
Ccnd1 B5 4	aaaacttcctcttcgcacttATTTCACTTCATATCACTCACTCCCAATCTCTATCTACCC
Ccnd1 B5 5	gtccaaataattcatcgccaATTTCACTTCATATCACTCACTCCCAATCTCTATCTACCC
Ccnd1 B5 6	tttcttgaggggttcgaacgATTTCACTTCATATCACTCACTCCCAATCTCTATCTACCC
Ccnd1 B5 7	tagctccgagcaattgcaacATTTCACTTCATATCACTCACTCCCAATCTCTATCTACCC

Ccnd1 B5 8	ttttctgcggtcagaggaatATTTCACTTCATATCACTCACTCCCAATCTCTATCTACCC
Ccnd1 B5 9	ggagttgtcggtgtaaatgcATTTCACTTCATATCACTCACTCCCAATCTCTATCTACCC
Ccnd1 B5 10	catttgcagtaactcgtcggATTTCACTTCATATCACTCACTCCCAATCTCTATCTACCC
Ccnd1 B5 11	aatgaaatcgtggggggtcaATTTCACTTCATATCACTCACTCCCAATCTCTATCTACCC
Ccnd1 B5 12	agaggcattttagtgaggaaATTTCACTTCATATCACTCACTCCCAATCTCTATCTACCC
Ccnd1 B5 13	gatgatctgtttggtgtcctATTTCACTTCATATCACTCACTCCCAATCTCTATCTACCC
Ccnd1 B5 14	aatttaacatctgtagcgcaATTTCACTTCATATCACTCACTCCCAATCTCTATCTACCC
Ccnd1 B5 15	gatcatggaaggtgggtttgATTTCACTTCATATCACTCACTCCCAATCTCTATCTACCC
Ccnd1 B5 16	aggaaagtgttagtgttcccATTTCACTTCATATCACTCACTCCCAATCTCTATCTACCC
Ccnd1 B5 17	acaatccggatcacatttgaATTTCACTTCATATCACTCACTCCCAATCTCTATCTACCC
Ccnd1 B5 18	ttcaatctgttcttggcaggATTTCACTTCATATCACTCACTCCCAATCTCTATCTACCC
Ccnd1 B5 19	tggcgtagactggattcaagATTTCACTTCATATCACTCACTCCCAATCTCTATCTACCC
Ccnd1 B5 20	caggaaaggtctgcttcgtcATTTCACTTCATATCACTCACTCCCAATCTCTATCTACCC
Ccnd1 B5 21	aatgttcacatctcgcacatATTTCACTTCATATCACTCACTCCCAATCTCTATCTACCC

Hyb 6

Runx2 B1 1	ttgctgacatggtgtcacggTATAGCATTCTTTCTTGAGGAGGGCAGCAAACGGGAAGAG
Runx2 B1 2	tggtgctgggatcccaaaagTATAGCATTCTTTCTTGAGGAGGGCAGCAAACGGGAAGAG
Runx2 B1 3	cgatgatctccaccatggtgTATAGCATTCTTTCTTGAGGAGGGCAGCAAACGGGAAGAG
Runx2 B1 4	cttcatcacagcagaagcatTATAGCATTCTTTCTTGAGGAGGGCAGCAAACGGGAAGAG
Runx2 B1 5	cacgaatcgcaggtcattgaTATAGCATTCTTTCTTGAGGAGGGCAGCAAACGGGAAGAG
Runx2 B1 6	gggatttgtcaggacagttaTATAGCATTCTTTCTTGAGGAGGGCAGCAAACGGGAAGAG
Runx2 B1 7	agagtcatcaagcttctgtcTATAGCATTCTTTCTTGAGGAGGGCAGCAAACGGGAAGAG
Runx2 B1 8	aggcgttcagggaacaaactTATAGCATTCTTTCTTGAGGAGGGCAGCAAACGGGAAGAG
Runx2 B1 9	ttggtgcagagttcagagagTATAGCATTCTTTCTTGAGGAGGGCAGCAAACGGGAAGAG
Runx2 B1 10	actctgtccttgtggattaaTATAGCATTCTTTCTTGAGGAGGGCAGCAAACGGGAAGAG
Runx2 B1 11	cgactgatcataggaccacgTATAGCATTCTTTCTTGAGGAGGGCAGCAAACGGGAAGAG
Runx2 B1 12	tcatctggctcaagtaggacTATAGCATTCTTTCTTGAGGAGGGCAGCAAACGGGAAGAG
Runx2 B1 13	gagtcgtggagtgaatggacTATAGCATTCTTTCTTGAGGAGGGCAGCAAACGGGAAGAG
Runx2 B1 14	gaactgcctgggatctgaaaTATAGCATTCTTTCTTGAGGAGGGCAGCAAACGGGAAGAG
Runx2 B1 15	gatagtgcattcgtgggttgTATAGCATTCTTTCTTGAGGAGGGCAGCAAACGGGAAGAG
Runx2 B1 16	cgggcggtgtataggtaaagTATAGCATTCTTTCTTGAGGAGGGCAGCAAACGGGAAGAG
Runx2 B1 17	cggacatacccagtgacatgTATAGCATTCTTTCTTGAGGAGGGCAGCAAACGGGAAGAG
Runx2 B1 18	caggtaggtgtggtagtgagTATAGCATTCTTTCTTGAGGAGGGCAGCAAACGGGAAGAG
Runx2 B1 19	actttggttttgggaggagcTATAGCATTCTTTCTTGAGGAGGGCAGCAAACGGGAAGAG
Runx2 B1 20	gacgaagtgccatagtagagTATAGCATTCTTTCTTGAGGAGGGCAGCAAACGGGAAGAG
Runx2 B1 21	caccatggggaactggtacgTATAGCATTCTTTCTTGAGGAGGGCAGCAAACGGGAAGAG
Runx2 B1 22	cggaggaagcatcctggaagTATAGCATTCTTTCTTGAGGAGGGCAGCAAACGGGAAGAG
Runx2 B1 23	caaaacagttggggagctgcTATAGCATTCTTTCTTGAGGAGGGCAGCAAACGGGAAGAG
Mitf B2 1	tagcaggattcgtgaggtcaAAAAAGCTCAGTCCATCCTCGTAAATCCTCATCAATCATC
Mitf B2 2	tgcatctgctcacgcatgagAAAAAGCTCAGTCCATCCTCGTAAATCCTCATCAATCATC
Mitf B2 3	tgacgttgatggcaggtgtcAAAAAGCTCAGTCCATCCTCGTAAATCCTCATCAATCATC
Mitf B2 4	gttggattctcaaggtgagtAAAAAGCTCAGTCCATCCTCGTAAATCCTCATCAATCATC
Mitf B2 5	ttggcgtgtttatttgctagAAAAAGCTCAGTCCATCCTCGTAAATCCTCATCAATCATC
Mitf B2 6	tcaagccaaggatttcttcaAAAAAGCTCAGTCCATCCTCGTAAATCCTCATCAATCATC
Mitf B2 7	tatttgccatttgcaaggctAAAAAGCTCAGTCCATCCTCGTAAATCCTCATCAATCATC
Mitf B2 8	atcaaattcccagacactggAAAAAGCTCAGTCCATCCTCGTAAATCCTCATCAATCATC
Mitf B2 9	catgctttggttgccataaaAAAAAGCTCAGTCCATCCTCGTAAATCCTCATCAATCATC
Mitf B2 10	tgctgatgttaagtcctggaAAAAAGCTCAGTCCATCCTCGTAAATCCTCATCAATCATC
Mitf B2 11	ttaggaagattagctggacaAAAAAGCTCAGTCCATCCTCGTAAATCCTCATCAATCATC
Mitf B2 12	ctcgcttctgactctgtgagAAAAAGCTCAGTCCATCCTCGTAAATCCTCATCAATCATC
Mitf B2 13	gttcttttatacggtcattaAAAAAGCTCAGTCCATCCTCGTAAATCCTCATCAATCATC
Mitf B2 14	cttattccagcgcatatccgAAAAAGCTCAGTCCATCCTCGTAAATCCTCATCAATCATC
Mitf B2 15	tgttctcaagttccttcgtgAAAAAGCTCAGTCCATCCTCGTAAATCCTCATCAATCATC
Mitf B2 16	ttggcgtgttccaatttcttAAAAAGCTCAGTCCATCCTCGTAAATCCTCATCAATCATC
Mitf B2 17	ctcaagttcctgtattctgaAAAAAGCTCAGTCCATCCTCGTAAATCCTCATCAATCATC
Mitf B2 18	accatatcaggggagcaaatAAAAAGCTCAGTCCATCCTCGTAAATCCTCATCAATCATC
Mitf B2 19	gcacaggttcttgtttgatgAAAAAGCTCAGTCCATCCTCGTAAATCCTCATCAATCATC
Mitf B2 20	ttccgaggttgtcactgaagAAAAAGCTCAGTCCATCCTCGTAAATCCTCATCAATCATC

Mitf B2 21	acactgtaagtgccagttggAAAAAGCTCAGTCCATCCTCGTAAATCCTCATCAATCATC
Mitf B2 22	cagtttggatcccatttttgAAAAAGCTCAGTCCATCCTCGTAAATCCTCATCAATCATC
Mitf B2 23	agggtatcgtccatcaagatAAAAAGCTCAGTCCATCCTCGTAAATCCTCATCAATCATC
Mitf B2 24	aacaagcatgatcagtgtccAAAAAGCTCAGTCCATCCTCGTAAATCCTCATCAATCATC
Plp1 B3 1	tatgagacagcgggcacagTAAAAAAGTCTAATCCGTCCCTGCCTCTATATCTCCACTC
Plp1 B3 2	cgaccagagaggcgaagggTAAAAAAGTCTAATCCGTCCCTGCCTCTATATCTCCACTC
Plp1 B3 3	cgaccccaaagaagcagagTAAAAAAGTCTAATCCGTCCCTGCCTCTATATCTCCACTC
Plp1 B3 4	taggtctcaatgagctgctTAAAAAAGTCTAATCCGTCCCTGCCTCTATATCTCCACTC
Plp1 B3 5	gctgttccatagatgacgtTAAAAAAGTCTAATCCGTCCCTGCCTCTATATCTCCACTC
Plp1 B3 6	tgtagaagccttcggccagTAAAAAAGTCTAATCCGTCCCTGCCTCTATATCTCCACTC
Plp1 B3 7	aagatttgccggactgcgcTAAAAAAGTCTAATCCGTCCCTGCCTCTATATCTCCACTC
Plp1 B3 8	cagatggtggtccggtagtTAAAAAAGTCTAATCCGTCCCTGCCTCTATATCTCCACTC
Plp1 B3 9	cagttaccgttgcgctgagTAAAAAAGTCTAATCCGTCCCTGCCTCTATATCTCCACTC
Plp1 B3 10	aaagagtgagctcgctgggTAAAAAAGTCTAATCCGTCCCTGCCTCTATATCTCCACTC
Plp1 B3 11	tcccaaacactgacacaccTAAAAAAGTCTAATCCGTCCCTGCCTCTATATCTCCACTC
Plp1 B3 12	acttgtcaggatgtcctagTAAAAAAGTCTAATCCGTCCCTGCCTCTATATCTCCACTC
Plp1 B3 13	cgatggtcaggacataggtTAAAAAAGTCTAATCCGTCCCTGCCTCTATATCTCCACTC
Plp1 B3 14	gaaggcgatggactggcagTAAAAAAGTCTAATCCGTCCCTGCCTCTATATCTCCACTC
Plp1 B3 15	gatgctggcagtggtcttgTAAAAAAGTCTAATCCGTCCCTGCCTCTATATCTCCACTC
Plp1 B3 16	caggacaccgtacatcctgTAAAAAAGTCTAATCCGTCCCTGCCTCTATATCTCCACTC
Plp1 B3 17	acacaccttcccgggaaacTAAAAAAGTCTAATCCGTCCCTGCCTCTATATCTCCACTC
Plp1 B3 18	agtcatttggaactcgctgTAAAAAAGTCTAATCCGTCCCTGCCTCTATATCTCCACTC
Plp1 B3 19	cagtgagaccagagtggcgTAAAAAAGTCTAATCCGTCCCTGCCTCTATATCTCCACTC
Plp1 B3 20	agtggcggcgatcatgaagTAAAAAAGTCTAATCCGTCCCTGCCTCTATATCTCCACTC
Col2A1 B4 1	catctttaatatctccgggtATTTCACATTTACAGACCTCAACCTACCTCCAACTCTCAC
Col2A1 B4 2	ttgaaatccctggggaccagATTTCACATTTACAGACCTCAACCTACCTCCAACTCTCAC
Col2A1 B4 3	gtgaaccagattcacccttgATTTCACATTTACAGACCTCAACCTACCTCCAACTCTCAC
Col2A1 B4 4	tgggagacccacaataccacATTTCACATTTACAGACCTCAACCTACCTCCAACTCTCAC
Col2A1 B4 5	ttttccagtgggaccaacagATTTCACATTTACAGACCTCAACCTACCTCCAACTCTCAC
Col2A1 B4 6	tagagccatctttgccagagATTTCACATTTACAGACCTCAACCTACCTCCAACTCTCAC
Col2A1 B4 7	cagtccagcaaaagcagacaATTTCACATTTACAGACCTCAACCTACCTCCAACTCTCAC
Col2A1 B4 8	gatttgagggtggcatccacATTTCACATTTACAGACCTCAACCTACCTCCAACTCTCAC
Col2A1 B4 9	cggatgctctcaatctgattATTTCACATTTACAGACCTCAACCTACCTCCAACTCTCAC
Col2A1 B4 10	gatggcagagtttgatgtcgATTTCACATTTACAGACCTCAACCTACCTCCAACTCTCAC
Col2A1 B4 11	ttcgggtcaatccagtaatcATTTCACATTTACAGACCTCAACCTACCTCCAACTCTCAC
Col2A1 B4 12	atactttgatggcgtccaagATTTCACATTTACAGACCTCAACCTACCTCCAACTCTCAC
Col2A1 B4 13	ggtctctgcaaaccagacgtATTTCACATTTACAGACCTCAACCTACCTCCAACTCTCAC
Col2A1 B4 14	agctgaagtggaaaccgccgATTTCACATTTACAGACCTCAACCTACCTCCAACTCTCAC
Col2A1 B4 15	atgaggatggctttcttcagATTTCACATTTACAGACCTCAACCTACCTCCAACTCTCAC
Col2A1 B4 16	tgatctccacgtcgttggatATTTCACATTTACAGACCTCAACCTACCTCCAACTCTCAC
Col2A1 B4 17	caagacgctgtaggtgaaccATTTCACATTTACAGACCTCAACCTACCTCCAACTCTCAC
Col2A1 B4 18	atttgccagtgtgtttcgtgATTTCACATTTACAGACCTCAACCTACCTCCAACTCTCAC
Col2A1 B4 19	tgcaaccggtactcgatcacATTTCACATTTACAGACCTCAACCTACCTCCAACTCTCAC
Col2A1 B4 20	aatgtccataggtgcagtatATTTCACATTTACAGACCTCAACCTACCTCCAACTCTCAC
HuD B5 1	ctccatggtgctaattatcaATTTCACTTCATATCACTCACTCCCAATCTCTATCTACCC
HuD B5 2	gtccattggttgtattggaaATTTCACTTCATATCACTCACTCCCAATCTCTATCTACCC
HuD B5 3	aagggcagtttctactgttgATTTCACTTCATATCACTCACTCCCAATCTCTATCTACCC
HuD B5 4	catgttctggggtaaatagtATTTCACTTCATATCACTCACTCCCAATCTCTATCTACCC
HuD B5 5	gattttgtctctcacgagctATTTCACTTCATATCACTCACTCCCAATCTCTATCTACCC
HuD B5 6	atccatatcctaaactctgcATTTCACTTCATATCACTCACTCCCAATCTCTATCTACCC
HuD B5 7	gcatcctttggatcaatgtaATTTCACTTCATATCACTCACTCCCAATCTCTATCTACCC
HuD B5 8	tctgagtccatttaaagtgtATTTCACTTCATATCACTCACTCCCAATCTCTATCTACCC
HuD B5 9	gatacctttatggttttggtATTTCACTTCATATCACTCACTCCCAATCTCTATCTACCC
HuD B5 10	ttgatgctgaacttggacggATTTCACTTCATATCACTCACTCCCAATCTCTATCTACCC
HuD B5 11	cgcccatattgtgagaataaATTTCACTTCATATCACTCACTCCCAATCTCTATCTACCC
HuD B5 12	cttgatcaaccagaatccgtATTTCACTTCATATCACTCACTCCCAATCTCTATCTACCC
HuD B5 13	taaatcccacacctcgagaaATTTCACTTCATATCACTCACTCCCAATCTCTATCTACCC
HuD B5 14	gcctctattctcttatcaaaATTTCACTTCATATCACTCACTCCCAATCTCTATCTACCC
HuD B5 15	aggatagcgtctgttgggagATTTCACTTCATATCACTCACTCCCAATCTCTATCTACCC

HuD B5 16	ggccatattaagcaaattgtATTTCACTTCATATCACTCACTCCCAATCTCTATCTACCC
HuD B5 17	ttggggagaacctcttaacgATTTCACTTCATATCACTCACTCCCAATCTCTATCTACCC
HuD B5 18	tgtgaccagggatattcattATTTCACTTCATATCACTCACTCCCAATCTCTATCTACCC
HuD B5 19	acagcacactctcatctgagATTTCACTTCATATCACTCACTCCCAATCTCTATCTACCC
HuD B5 20	attattgaccgctcccgaagATTTCACTTCATATCACTCACTCCCAATCTCTATCTACCC
HuD B5 21	aaagccaaatcccttgcactATTTCACTTCATATCACTCACTCCCAATCTCTATCTACCC
HuD B5 22	atccattaaggctggcaatcATTTCACTTCATATCACTCACTCCCAATCTCTATCTACCC
HuD B5 23	tgcaatactctgtctcctaaATTTCACTTCATATCACTCACTCCCAATCTCTATCTACCC
HuD B5 24	ggatttgtgggttttattggATTTCACTTCATATCACTCACTCCCAATCTCTATCTACCC

Hyb 7

Krt14 B1 1	gaactgccggacagtggtgTATAGCATTCTTTCTTGAGGAGGGCAGCAAACGGGAAGAG
Krt14 B1 2	cttgagggaggtggaggagTATAGCATTCTTTCTTGAGGAGGGCAGCAAACGGGAAGAG
Krt14 B1 3	agacgatgcgggaggagacTATAGCATTCTTTCTTGAGGAGGGCAGCAAACGGGAAGAG
Krt14 B1 4	cattgtttccttctcgccgTATAGCATTCTTTCTTGAGGAGGGCAGCAAACGGGAAGAG
Krt14 B1 5	tgatcttcacctccagatcTATAGCATTCTTTCTTGAGGAGGGCAGCAAACGGGAAGAG
Krt14 B1 6	aagaatcttgctgcgcagcTATAGCATTCTTTCTTGAGGAGGGCAGCAAACGGGAAGAG
Krt14 B1 7	ggcattctcaacagttgcgTATAGCATTCTTTCTTGAGGAGGGCAGCAAACGGGAAGAG
Krt14 B1 8	ctgctccgtctcaaacttgTATAGCATTCTTTCTTGAGGAGGGCAGCAAACGGGAAGAG
Krt14 B1 9	tctcaatctgcatctccagTATAGCATTCTTTCTTGAGGAGGGCAGCAAACGGGAAGAG
Krt14 B1 10	tgaggtcaatgccgggagcTATAGCATTCTTTCTTGAGGAGGGCAGCAAACGGGAAGAG
Krt14 B1 11	gcatctcagccaggatcttTATAGCATTCTTTCTTGAGGAGGGCAGCAAACGGGAAGAG
Krt14 B1 12	gaagaaccactgctcagcaTATAGCATTCTTTCTTGAGGAGGGCAGCAAACGGGAAGAG
Krt14 B1 13	gatctccaggctctggatgTATAGCATTCTTTCTTGAGGAGGGCAGCAAACGGGAAGAG
Krt14 B1 14	catgtcacagcgcagctcgTATAGCATTCTTTCTTGAGGAGGGCAGCAAACGGGAAGAG
Krt14 B1 15	actgggaagagatgtgggcTATAGCATTCTTTCTTGAGGAGGGCAGCAAACGGGAAGAG
Krt14 B1 16	gaggtcgtaatggcgtctcTATAGCATTCTTTCTTGAGGAGGGCAGCAAACGGGAAGAG
Nestin B2 1	ctctggatctcagctcgaagAAAAAGCTCAGTCCATCCTCGTAAATCCTCATCAATCATC
Nestin B2 2	tctctgacagtcttttggagAAAAAGCTCAGTCCATCCTCGTAAATCCTCATCAATCATC
Nestin B2 3	gacatcttgaggtgcatgagAAAAAGCTCAGTCCATCCTCGTAAATCCTCATCAATCATC
Nestin B2 4	cgagctgattttctggaactAAAAAGCTCAGTCCATCCTCGTAAATCCTCATCAATCATC
Nestin B2 5	atcttgcatctccttgagagAAAAAGCTCAGTCCATCCTCGTAAATCCTCATCAATCATC
Nestin B2 6	tgagtgtgggcacttctttgAAAAAGCTCAGTCCATCCTCGTAAATCCTCATCAATCATC
Nestin B2 7	tctgggtggcacttaacaatAAAAAGCTCAGTCCATCCTCGTAAATCCTCATCAATCATC
Nestin B2 8	agcacaagcagcttcgatttAAAAAGCTCAGTCCATCCTCGTAAATCCTCATCAATCATC
Nestin B2 9	ggattcctgttggcaaaactAAAAAGCTCAGTCCATCCTCGTAAATCCTCATCAATCATC
Nestin B2 10	cttcaatgctagaagccttcAAAAAGCTCAGTCCATCCTCGTAAATCCTCATCAATCATC
Nestin B2 11	tcctgaaggtctctatgttcAAAAAGCTCAGTCCATCCTCGTAAATCCTCATCAATCATC
Nestin B2 12	cttgctgcttttgactgatgAAAAAGCTCAGTCCATCCTCGTAAATCCTCATCAATCATC
Nestin B2 13	tctgcatgtgggttgtcatcAAAAAGCTCAGTCCATCCTCGTAAATCCTCATCAATCATC
Nestin B2 14	tcaatgggttctgagctgagAAAAAGCTCAGTCCATCCTCGTAAATCCTCATCAATCATC
Nestin B2 15	agcatcctctgactgaaagtAAAAAGCTCAGTCCATCCTCGTAAATCCTCATCAATCATC
Nestin B2 16	tgtatgcacatagcggcgtgAAAAAGCTCAGTCCATCCTCGTAAATCCTCATCAATCATC
Nestin B2 17	actataaagtacccttcctcAAAAAGCTCAGTCCATCCTCGTAAATCCTCATCAATCATC
Nestin B2 18	ctgttagttcatcctttctgAAAAAGCTCAGTCCATCCTCGTAAATCCTCATCAATCATC
Nestin B2 19	caccaaagatgctactccagAAAAAGCTCAGTCCATCCTCGTAAATCCTCATCAATCATC
Nestin B2 20	tcctttggcatctcaaattcAAAAAGCTCAGTCCATCCTCGTAAATCCTCATCAATCATC
Nestin B2 21	agggtggtcttatcagtgtgAAAAAGCTCAGTCCATCCTCGTAAATCCTCATCAATCATC
Nestin B2 22	cagtgttggagggatcagtgAAAAAGCTCAGTCCATCCTCGTAAATCCTCATCAATCATC
Nestin B2 23	acatcggggagatattcagcAAAAAGCTCAGTCCATCCTCGTAAATCCTCATCAATCATC
Nestin B2 24	tatctccagaatatccgagtAAAAAGCTCAGTCCATCCTCGTAAATCCTCATCAATCATC
FabP7 B3 1	cttccacgtcgcgcagaaagTAAAAAAGTCTAATCCGTCCCTGCCTCTATATCTCCACTC
FabP7 B3 2	gccttcatgtattcgtcaaaTAAAAAAGTCTAATCCGTCCCTGCCTCTATATCTCCACTC
FabP7 B3 3	gatgatcactgtgggcttagTAAAAAAGTCTAATCCGTCCCTGCCTCTATATCTCCACTC
FabP7 B3 4	agtcctgatcacgactttgtTAAAAAAGTCTAATCCGTCCCTGCCTCTATATCTCCACTC
FabP7 B3 5	tcgccgagtttaaagctgatTAAAAAAGTCTAATCCGTCCCTGCCTCTATATCTCCACTC
FabP7 B3 6	gggggtggtttcatcaaattTAAAAAAGTCTAATCCGTCCCTGCCTCTATATCTCCACTC
FabP7 B3 7	cacaactgatttgcagttccTAAAAAAGTCTAATCCGTCCCTGCCTCTATATCTCCACTC
FabP7 B3 8	cccatttctgtacatgaactTAAAAAAGTCTAATCCGTCCCTGCCTCTATATCTCCACTC

FabP7 B3 9	acaaagtttgtctctttgccTAAAAAAGTCTAATCCGTCCCTGCCTCTATATCTCCACTC
FabP7 B3 10	attctgccatcctttatttcTAAAAAAGTCTAATCCGTCCCTGCCTCTATATCTCCACTC
FabP7 B3 11	caccacatcaccaaaggtaaTAAAAAAGTCTAATCCGTCCCTGCCTCTATATCTCCACTC
FabP7 B3 12	ctttctcatagtggcgaacaTAAAAAAGTCTAATCCGTCCCTGCCTCTATATCTCCACTC
Krt19 B4 1	ctgaagctataagtcgccatATTTCACATTTACAGACCTCAACCTACCTCCAACTCTCAC
Krt19 B4 2	tggcggaagagactgacactATTTCACATTTACAGACCTCAACCTACCTCCAACTCTCAC
Krt19 B4 3	cctaggccagaagagacaaaATTTCACATTTACAGACCTCAACCTACCTCCAACTCTCAC
Krt19 B4 4	agaggagaccgtcaccagagATTTCACATTTACAGACCTCAACCTACCTCCAACTCTCAC
Krt19 B4 5	atcgttgtcttttcattcccATTTCACATTTACAGACCTCAACCTACCTCCAACTCTCAC
Krt19 B4 6	tctgagtttgcttcttccaaATTTCACATTTACAGACCTCAACCTACCTCCAACTCTCAC
Krt19 B4 7	ctcccggattttagtttcaaATTTCACATTTACAGACCTCAACCTACCTCCAACTCTCAC
Krt19 B4 8	aataagggctgtagtctcggATTTCACATTTACAGACCTCAACCTACCTCCAACTCTCAC
Krt19 B4 9	cggaggtcttcaatagccttATTTCACATTTACAGACCTCAACCTACCTCCAACTCTCAC
Krt19 B4 10	tagtggcagcaaggatctggATTTCACATTTACAGACCTCAACCTACCTCCAACTCTCAC
Krt19 B4 11	cttggtcttgaagtcgtcagATTTCACATTTACAGACCTCAACCTACCTCCAACTCTCAC
Krt19 B4 12	gaagagcttgctctgtttcaATTTCACATTTACAGACCTCAACCTACCTCCAACTCTCAC
Krt19 B4 13	atactggtctctcatatcggATTTCACATTTACAGACCTCAACCTACCTCCAACTCTCAC
Krt19 B4 14	cagtcttactgtggaaccagATTTCACATTTACAGACCTCAACCTACCTCCAACTCTCAC
Krt19 B4 15	tgtagctgttcggtattgacATTTCACATTTACAGACCTCAACCTACCTCCAACTCTCAC
Krt19 B4 16	cagtgacttcagacttgctgATTTCACATTTACAGACCTCAACCTACCTCCAACTCTCAC
Krt19 B4 17	ttcatgctgagctgggactgATTTCACATTTACAGACCTCAACCTACCTCCAACTCTCAC
Krt19 B4 18	tcaatgctgccaatcatgtcATTTCACATTTACAGACCTCAACCTACCTCCAACTCTCAC
Krt19 B4 19	atcttgtattcgctgttctgATTTCACATTTACAGACCTCAACCTACCTCCAACTCTCAC
Krt19 B4 20	acgggtcttgatatccatgaATTTCACATTTACAGACCTCAACCTACCTCCAACTCTCAC
Krt19 B4 21	taagtagcgatctcctgctcATTTCACATTTACAGACCTCAACCTACCTCCAACTCTCAC
Krt19 B4 22	ttttgtcgggagatccagaaATTTCACATTTACAGACCTCAACCTACCTCCAACTCTCAC
Alx1 B5 1	tgctcccatgtaaaagtcacATTTCACTTCATATCACTCACTCCCAATCTCTATCTACCC
Alx1 B5 2	ggactcattgtccaaagtttATTTCACTTCATATCACTCACTCCCAATCTCTATCTACCC
Alx1 B5 3	tgcctgacgttttgctataaATTTCACTTCATATCACTCACTCCCAATCTCTATCTACCC
Alx1 B5 4	ttgaaggcctgcacacatttATTTCACTTCATATCACTCACTCCCAATCTCTATCTACCC
Alx1 B5 5	catagttcacgttgttgtctATTTCACTTCATATCACTCACTCCCAATCTCTATCTACCC
Alx1 B5 6	ccagctgcaaactggtgaaaATTTCACTTCATATCACTCACTCCCAATCTCTATCTACCC
Alx1 B5 7	ctcacgtagacatcagggtaATTTCACTTCATATCACTCACTCCCAATCTCTATCTACCC
Alx1 B5 8	ctcttagcttgctggatctgATTTCACTTCATATCACTCACTCCCAATCTCTATCTACCC
Alx1 B5 9	atatatcataagtggcagcaATTTCACTTCATATCACTCACTCCCAATCTCTATCTACCC
Alx1 B5 10	ctgtcagtccttggaagaacATTTCACTTCATATCACTCACTCCCAATCTCTATCTACCC
Alx1 B5 11	acagaaccactagcggtgttATTTCACTTCATATCACTCACTCCCAATCTCTATCTACCC
Alx1 B5 12	gtggtatcatgcaggaagtaATTTCACTTCATATCACTCACTCCCAATCTCTATCTACCC
Alx1 B5 13	ccggggtgaatgggaataagATTTCACTTCATATCACTCACTCCCAATCTCTATCTACCC
Alx1 B5 14	aagcctgtgtagccagaatcATTTCACTTCATATCACTCACTCCCAATCTCTATCTACCC
Alx1 B5 15	gaactgattctggtggtttgATTTCACTTCATATCACTCACTCCCAATCTCTATCTACCC
Alx1 B5 16	aattattgagaggcacatggATTTCACTTCATATCACTCACTCCCAATCTCTATCTACCC
Alx1 B5 17	ccccagaaagtaaagagtcaATTTCACTTCATATCACTCACTCCCAATCTCTATCTACCC
Alx1 B5 18	tcaaaagcatgtccattggtATTTCACTTCATATCACTCACTCCCAATCTCTATCTACCC
Alx1 B5 19	tcctttcaaattccggcttgATTTCACTTCATATCACTCACTCCCAATCTCTATCTACCC
Alx1 B5 20	agaactgcaatgctggaagaATTTCACTTCATATCACTCACTCCCAATCTCTATCTACCC
Alx1 B5 21	atagcccaggaaatattggcATTTCACTTCATATCACTCACTCCCAATCTCTATCTACCC

Supplementary Table 1

List of probes used for the SGA analysis Each probe consists of a consensus sequence complementary to mRNA (lowercase letters), as well as a linker sequence and an initiator sequence (B1-B5) for the hairpins used for HCR amplification (capital letters).
