# Supplementary material for: Identification of a neural crest stem cell niche by Spatial Genomic Analysis
Source: Nat Commun. 2017 Nov 28;8:1830. doi: 10.1038/s41467-017-01561-w (PMC5705662; doi:10.1038/s41467-017-01561-w)
Supplement: Supplementary file 2 — Description of Additional Supplementary Files [file 41467_2017_1561_MOESM2_ESM.docx]

**Description of Additional Supplementary Files**

File Name: Supplementary Movie 1

Description: **A scroll trough of z-stacks (0.5 μm each) visualizing machine learning algorithm based automated cell segmentation.** Left panel: Raw image of the plasma membranes immunostained with antibodies (E-cadherin and β-catenin). Right panel: computer generated cell boundaries overlaying the raw image. Cells that are accepted for the analysis are filtered based of Gaussian distribution of their volume.
